# Targeting myeloid-derived suppressor cells in combination with primary mammary tumor resection reduces metastatic growth in the lungs

**DOI:** 10.1186/s13058-019-1189-x

**Published:** 2019-09-05

**Authors:** Momir Bosiljcic, Rachel A. Cederberg, Melisa J. Hamilton, Nancy E. LePard, Bryant T. Harbourne, Jenna L. Collier, Elizabeth C. Halvorsen, Rocky Shi, S. Elizabeth Franks, Ada Y. Kim, Judit P. Banáth, Mark Hamer, Fabio M. Rossi, Kevin L. Bennewith

**Affiliations:** 1 Integrative Oncology Department, BC Cancer Research Centre, Room 10-108, 675 West 10th Avenue, Vancouver, British Columbia V5Z 1L3 Canada; 2 Pathology and Laboratory Medicine, University of British Columbia, Vancouver, British Columbia Canada; 3 Interdisciplinary Oncology Program, University of British Columbia, Vancouver, British Columbia Canada; 4 Biomedical Research Centre, University of British Columbia, 2222 Health Sciences Mall, Vancouver, British Columbia V6T 1Z3 Canada

**Keywords:** Myeloid-derived suppressor cells, Macrophages, Eosinophils, Metastasis, Tumor resection, Gemcitabine, 4T1, 4T07, 67NR

## Abstract

**Background:**

Solid tumors produce proteins that can induce the accumulation of bone marrow-derived cells in various tissues, and these cells can enhance metastatic tumor growth by several mechanisms. 4T1 murine mammary tumors are known to produce granulocyte colony-stimulating factor (G-CSF) and increase the numbers of immunosuppressive CD11b^+^Gr1^+^ myeloid-derived suppressor cells (MDSCs) in tissues such as the spleen and lungs of tumor-bearing mice. While surgical resection of primary tumors decreases MDSC levels in the spleen, the longevity and impact of MDSCs and other immune cells in the lungs after tumor resection have been less studied.

**Methods:**

We used mass cytometry time of flight (CyTOF) and flow cytometry to quantify MDSCs in the spleen, peripheral blood, and lungs of mice bearing orthotopic murine mammary tumors. We also tested the effect of primary tumor resection and/or gemcitabine treatment on the levels of MDSCs, other immune suppressor and effector cells, and metastatic tumor cells in the lungs.

**Results:**

We have found that, similar to mice with 4T1 tumors, mice bearing metastatic 4T07 tumors also exhibit accumulation of CD11b^+^Gr1^+^ MDSCs in the spleen and lungs, while tissues of mice with non-metastatic 67NR tumors do not contain MDSCs. Mice with orthotopically implanted 4T1 tumors have increased granulocytic (G-) MDSCs, monocytic (M-) MDSCs, macrophages, eosinophils, and NK cells in the lungs. Resection of primary 4T1 tumors decreases G-MDSCs, M-MDSCs, and macrophages in the lungs within 48 h, but significant numbers of functional immunosuppressive G-MDSCs persist in the lungs for 2 weeks after tumor resection, indicative of an environment that can promote metastatic tumor growth. The chemotherapeutic agent gemcitabine depletes G-MDSCs, M-MDSCs, macrophages, and eosinophils in the lungs of 4T1 tumor-bearing mice, and we found that treating mice with gemcitabine after primary tumor resection decreases residual G-MDSCs in the lungs and decreases subsequent metastatic growth.

**Conclusions:**

Our data support the development of therapeutic strategies to target MDSCs and to monitor MDSC levels before and after primary tumor resection to enhance the effectiveness of immune-based therapies and improve the treatment of metastatic breast cancer in the clinic.

**Electronic supplementary material:**

The online version of this article (10.1186/s13058-019-1189-x) contains supplementary material, which is available to authorized users.

## Background

An estimated 90% of cancer-related deaths are associated with tumor metastases [1, 2], highlighting the need for new and effective therapeutic strategies to treat metastatic disease. The contribution of host tissues to the development of metastatic tumor growth was first postulated by Stephen Paget in the late 1800s, with his “seed-and-soil” hypothesis [3] suggesting that metastatic tumor cells (seeds) must enter suitable host tissues (soil) in order to grow into tumor metastases. More recent evidence suggests that primary tumors can “fertilize” the metastatic soil within some tissues to promote metastatic tumor growth. Localized microenvironments can form in metastatic target organs, consisting of supportive stromal cells, pro-tumorigenic proteins, and a variety of different bone marrow-derived cells. These “pre-metastatic niches” are thought to represent fertile regions of tissue that facilitate the subsequent invasion, survival, and proliferation of metastatic tumor cells [4, 5]. Pre-metastatic niches develop prior to the arrival of metastatic tumor cells and can be induced by injection of tumor cell-derived conditioned medium into tumor-free mice [6–8]. Exosomes secreted by tumor cells can contribute to pre-metastatic niche development [9], providing a mechanism for the delivery of proteins from primary metastatic tumors to distant tissues [10, 11]. The tumor-derived factors and cells present in pre-metastatic niches differ between model tumor systems [5], and evidence in immunocompetent mice indicates an important role for immunosuppressive cells in promoting metastatic growth in distant tissues.

Bone marrow-derived cells have been detected in metastatic tissues of cancer patients [7, 8], and cells that express the cell surface marker CD11b may be particularly important for promoting breast cancer metastasis [7]. CD11b (Mac-1) is an α_M_ integrin expressed on a variety of myeloid cells (granulocytes, monocytes, and macrophages), natural killer (NK) cells, and a subset of B cells. CD11b^+^ myeloid cells, often co-expressing Gr1, are increased in some primary tumors and have been implicated in enhancing tumor cell invasion [12, 13], angiogenesis [14, 15], and vasculogenesis [16]. Granulocyte-colony stimulating factor (G-CSF) secreted by 4T1 mammary tumor cells has been shown to induce CD11b^+^Gr1^+^ cell expansion [17], and we, along with others, have shown that CD11b^+^Gr1^+^ cells accumulate in the spleens and lungs of mice bearing metastatic 4T1 murine mammary tumors [17–20]. CD11b^+^Gr1^+^ cells represent a heterogeneous mixture of myeloid cells, including neutrophils and myeloid-derived suppressor cells (MDSCs) [21].

MDSCs accumulate in response to inflammatory stimuli and normally function to prevent auto-immunity and resolve inflammation [22]. MDSCs can be distinguished from other CD11b^+^Gr1^+^ myeloid cells by their ability to inhibit T cell- and NK cell-mediated immune responses [22–24]. Aberrantly elevated levels of MDSCs have been described in tumor-bearing mice and cancer patients [25] and are thought to be important mediators of tumor development and progression by actively suppressing the activity of cytotoxic T cells. Two sub-types of MDSCs have been identified in mice [26], with CD11b^+^Ly6G^+^Ly6C^mid/lo^ granulocytic MDSCs (G-MDSCs) exhibiting less immunosuppressive potency than the less abundant CD11b^+^Ly6G^−^Ly6C^hi^ monocytic MDSCs (M-MDSCs) [22]. While the presence of CD11b^+^Gr1^+^ cells in the lungs has been associated with enhanced growth of metastatic tumor cell foci [17–19], CD11b^+^Gr1^+^ cells have also been shown to accumulate in non-metastatic target organs of tumor-bearing mice [27] and in the peripheral blood of breast cancer patients [28–30]. Primary tumor resection in mice is known to decrease MDSC levels in the spleen [31, 32], although the longevity of MDSCs in the lungs after primary tumor resection, and the potential impact of these MDSCs on metastatic growth in the lungs, is less well-understood.

We have found that in addition to mice bearing 4T1 tumors, mice orthotopically implanted with metastatic 4T07 murine mammary tumors, but not non-metastatic 67NR tumors, have high levels of functional, immunosuppressive CD11b^+^Gr1^+^ MDSCs in the lungs. In addition to MDSCs in the lungs of 4T1 tumor-bearing mice, we also found elevated inflammatory macrophages, eosinophils, and NK cells. G-MDSCs, M-MDSCs, and macrophages rapidly decrease in the lungs within 48 h of primary 4T1 tumor resection, although G-MDSCs in the lungs remain higher than naïve mice for 2 weeks following tumor resection. These residual pulmonary G-MDSCs retain immunosuppressive function and are associated with enhanced metastatic tumor cell colonization in the lungs, indicative of a pro-metastatic environment in lung tissue that persists after primary tumor resection. Treating mice with gemcitabine after surgery decreases residual G-MDSCs and tumor colonization of the lungs, suggesting that targeting MDSCs after primary tumor resection may improve the treatment of metastatic breast cancer.

## Methods

### Tumor cells and mice

4T1, 4T07, and 67NR murine mammary carcinoma cells were a kind gift from Dr. Fred Miller (Karmanos Cancer Institutes, Detroit, MI). These cell lines were originally derived from a spontaneous mammary tumor in a Balb/cfC3H mouse and represent different levels of metastatic propensity [33]. 4T1 tumor cells metastasize to the lung, liver, bone, and brain; 4T07 cells metastasize to the lungs and liver, but fail to grow into macroscopic metastases; 67NR cells do not metastasize. MSC2 cells are an immortalized MDSC cell line obtained from BALB/C Gr1^+^ splenocytes and were provided from Dr. François Ghiringhelli (University of Burgundy, Dijon, France). All cells were maintained in RPMI-1640 medium + sodium pyruvate, HEPES, and 10% FCS and used within 20 passages.

Female Balb/c mice (8–10 weeks old) were purchased from Simonsen Laboratories (Gilroy, CA). All mice were housed under specific pathogen-free conditions in the Animal Resource Centre at the BC Cancer Research Centre. All animal experiments were performed in accordance with Institutional and Canadian Council on Animal Care Guidelines. For orthotopic mammary tumor implantation, mice were injected with 10^5^ 4T1 cells, 10^6^ 4T07 cells, or 2 × 10^5^ 67NR cells in the fourth mammary fat pad. We have found that these cell numbers produce consistent tumor growth rates, with tumor volumes that approach ethical restrictions (1–1.25 cm^3^) 4 weeks after implantation. For the intravenous (iv) studies, mice were injected with 1.2 × 10^4^ 4T1 cells in a 200-μl injection volume of PBS into the lateral tail vein.

### Primary tumor resections

For resection of orthotopically implanted 4T1 tumors, mice were anesthetized with 2.5% isoflurane in oxygen prior to shaving over the left side of the body (adjacent to the tumor implantation site in the fourth mammary fat pad). The skin was scrubbed with 4% germi-stat and 70% alcohol prior to injection of 10–20 μL of 0.5% lidocaine as a subcutaneous line block along the intended incision site. A ~ 6-mm incision was made adjacent to the tumor using sterile surgical scissors. The blunt end of a trocar was used to gently separate the tumor from the overlying skin, and the tumor was gently pulled through the incision using sterilized forceps. After tumor resection, the incision site was closed with nylon monofilament non-absorbable sutures. Mice were injected with 5 mg/kg meloxicam into the dorsal neck pouch followed by 10 mL/kg warmed saline. Tumor-bearing mice exposed to sham surgery underwent all of the above procedures other than tumor resection; incisions were open for a total of 5–6 min per mouse, and mice received all anesthetics and analgesics.

### Gemcitabine and antibody treatments

A single dose of 60 mg/kg of gemcitabine (Sandoz, Boucherville, QC) was injected ip into mice bearing 17-day-old 4T1 tumors or 1 day following 4T1 primary tumor excision. Gemcitabine was diluted to a 15 mg/ml working solution in physiological saline. Mice were euthanized and tissues were harvested 24, 48, 72, and 96 h post drug administration.

For immunological depletion of MDSCs, 100 μg of anti-Gr1 antibody (clone 1A8; BioXCell) or IgG2b isotype control (clone LTF-2; BioXCell) was administered via ip injection or intranasally every 4 days beginning 7 days after primary tumor implant. We found that 200 μg of anti-Gr1 antibody was lethal by the third injection. For immunological depletion of eosinophils, anti-IL-5 antibody (TRFK5, BioXCell) or isotype control (TNP6A7, BioXCell) was administered by weekly ip injection at 1 mg/kg in PBS.

### Blood processing

For analysis of circulating CD11b^+^Gr1^+^ cells, 100 μl of murine peripheral blood was collected once per week by inserting a 26-G needle into the lateral tail vein to collect blood into a heparin-coated capillary tube. Blood samples were transferred to K_2_EDTA-treated tubes (BD Microtainer, Franklin Lakes, NJ), centrifuged at 1000×*g* for 10 min at room temperature, and the plasma removed. The cellular fraction of each sample was treated with NH_4_Cl for 9 min on ice to induce erythrocyte lysis prior to antibody incubation for subsequent flow cytometry analyses. For antibody array or enzyme-linked immunosorbent assay (ELISA) analyses, plasma was collected by terminal cardiac puncture using a heparin-coated syringe with a 26-G needle prior to processing as outlined above.

### Antibody array and mG-CSF quantification

Plasma was collected from naïve and 4T1 tumor-bearing mice as previously described, and chemokines were analyzed with an R&D Systems Mouse Cytokine Array, Panel A (Catalog # ARY006) according to the manufacturer’s instructions. Array images were developed onto X-ray film and digitized with a flatbed scanner.

G-CSF serum levels were quantified using a mouse G-CSF Quantikine ELISA (R&D Systems, Minneapolis, MN) as per the manufacturer’s protocol. ELISA plates were analyzed using a Tecan Safire^2^ at 450 nm with wavelength correction at 540 nm.

### Tissue processing

The spleens and livers were pushed through 100-μm and 40-μm mesh filters with PBS to create single-cell suspensions. For clonogenic and immune suppression assays, lungs and kidneys were finely minced with crossed scalpels prior to agitation for 40 min at 37 °C with an enzyme suspension containing 0.5% trypsin and 0.08% collagenase I in PBS (for clonogenic assays). After incubation, 0.06% DNase was added and the cell suspension was gently vortexed and filtered through 30-μm nylon mesh. Single-cell suspensions were treated with NH_4_Cl for 9 min on ice to induce erythrocyte lysis. For flow cytometry analyses, lungs were processed as above except with 1 mg/mL collagenase II (Gibco Life Technologies) in RPMI medium for the tissue digestion step (no trypsin or DNase).

Clonogenic assays from disaggregated lung tissue were performed as previously reported [34, 35]. Briefly, single-cell suspensions derived from lung tissue were washed in PBS, and aliquots of 3 × 10^3^ to 10^6^ cells were plated in triplicate in medium containing 60 μM 6-thioguanine to select for the 6-thioguanine-resistant 4T1 tumor cells. Plates were incubated for 10–12 days prior to staining cell colonies with malachite green for manual enumeration.

### Mass cytometry

Antibody labeling with the indicated metal tag was performed using the MaxPAR antibody conjugation kit (Fluidigm), and concentration was assessed after metal conjugation using a Nanodrop (Thermo Scientific). Single-cell suspensions of lung cells were fixed with 1.6% paraformaldehyde (PFA; Electron Microscopy Sciences) for 10 min at room temperature. Cells were washed in PBS + 2% FBS and resuspended in blocking buffer (PBS + 5% FBS) and 1.5 μg/mL anti-mouse CD32 antibody at a concentration of 3 × 10^6^ cells/50 μL for 10 min. Cells were then stained for 45 min on ice with antibodies at a concentration of 3 × 10^6^ cells/100 μL. The cells were subsequently washed twice with MaxPar Cell Staining Buffer (Fluidigm) before being permeabilized and fixed by incubation in 1 mL of MaxPar Fix and Perm Buffer for 1.5 h. Cells were subsequently washed twice with MaxPar Perm-s Buffer and stained with intracellular antibody at 3 × 10^6^ cells/100 μL in MaxPar Perm-s Buffer before being washed twice with MaxPar Cell Staining Buffer (Millipore). EQ™ Four Element Calibration Beads (DVS Sciences) were added at a concentration of 3.3 × 10^4^ beads/mL to the cells in milli-Q H_2_O at a cell concentration of 1 × 10^6^ cells/mL. Cells were then filtered and run on a CyTOF 2 (Fluidigm) with a flow speed of 0.045 mL/min, a 30-s acquisition delay, and 10-s detector stability delay.

Data files were concatenated using the FCS file concatenation tool available from Cytobank (https://www.cytobank.org/) and normalized using software in MatLab (MathWorks) [36]. Normalized data was debarcoded using a debarcoding tool with cell and sample-specific stringency adjustment [37]. Data were analyzed in R using the package “cytofkit”: a total of 10,000 cells were downsampled from each sample without replacement for ArcSinh transformation and subsequent t-SNE analysis for PhenoGraph clustering and viSNE visualization. Other analyses were completed using FlowJo VX (Treestar). Cell surface markers used to identify each immune cell subset in the lungs are listed in Additional file 1: Table S1.

### T cell proliferation assay

Spleen or lung tissue of naïve mice or mice 3 weeks after primary mammary tumor implant were harvested and CD11b^+^Gr1^+^ cells were isolated from single-cell suspensions via Gr1-PE positive selection using the EasySep system (StemCell Technologies, Vancouver, BC, Canada) according to the manufacturer’s instructions. CD11b^+^Gr1^+^ cell purity of the isolated cells was > 95% as determined by subsequent flow cytometry analysis. Immunosuppression assays were performed using HL-1 medium (BioWhittaker; Basel, Switzerland), supplemented with 1% penicillin, 1% streptomycin, 1% Glutamax, and 50 μM 2-mercaptoethanol. We did not use serum in these assays, as we have previously found that the use of serum in immunosuppression assays can mask the immunosuppressive function of CD11b^+^Gr1^+^ cells [34]. Erythrocyte-depleted splenocytes (an abundant source of T cells) from naïve mice stimulated ex vivo with 1 μg/ml anti-CD3 + 5 μg/ml anti-CD28 (eBioscience, San Diego, CA) were used as responder cells in the assay and cultured at 2 × 10^5^ cells/well ± isolated CD11b^+^Gr1^+^ cells. Co-cultured cells were incubated at 37 °C for 72 h, and 1 μCi/well ^3^H-thymidine (2 Ci/mM, PerkinElmer, Woodbridge, ON, Canada) was added for the last 18 h of the assay. Cells were harvested onto filtermats, and radioactivity was measured using a Betaplate liquid scintillation counter (Wallac, Waltham, MA). Data are expressed as mean ± SEM of the ^3^H counts per minute (cpm) from triplicate cultures, or as cell proliferation relative to control samples (stimulated splenocytes alone).

### Flow cytometry

5 × 10^5^ freshly harvested cells (or rehydrated alcohol-fixed cells for BrdU analyses) were washed in PBS + 4% FCS prior to incubation with primary antibodies. Cells were stained with the following antibodies: CD11b-PE, Gr1-Alexa 488 (Invitrogen), Ly6G-PE, Ly6C-FITC (BD Pharmigen), and unconjugated F4/80 (eBioscience). When unconjugated F4/80 was used, cells were incubated with Alexa-488 or Alexa-594 secondary antibodies (Invitrogen). Where indicated, mice were given 90 mg/kg 5-bromo-2′-deoxyuridine (BrdU; Sigma-Aldrich, Oakville, ON) intraperitoneally (ip) 90 min before tissue harvest. For BrdU analysis, cells were denatured with HCl prior to neutralization and anti-BrdU (Abcam, Toronto, ON, Canada) contact in PBS + 4% FCS + 0.1% Triton-X. List mode files were collected using a dual laser Epics Elite-ESP flow cytometer (Coulter Corp., Hialeah, FL) and were subsequently reprocessed for analysis. Doublet correction and bitmap gating were used to select the cell populations of interest with the WINLIST software package (Verity Software House Inc., Topsham, ME).

Peripheral blood samples were stained with Fixable Viability Dye eFluor 780 (eBioscience, San Diego, CA) after NH_4_Cl treatment. In addition to CD11b-PE and Gr1-Alexa 488, blood samples were stained with CD45-APC (eBioscience, San Diego, CA), samples were run on a FACSCalibur (DxP 6-color Upgrade), and events were acquired/analyzed using FlowJo CE software. For cell cycle analysis data, ethanol fixed lung and spleen samples were rehydrated and 5 × 10^5^ to 1 × 10^6^ cells were stained with propidium iodide (PI) in order to generate DNA profiles. FlowJo CE software was used to analyze cell cycle profiles.

For 12–15 color flow cytometry panels, single-cell suspensions from the lungs were washed with PBS and stained for 30 min on ice with eFluor® 780 fixable-viability dye (eBioscience). Cells were washed and resuspended in Hanks balanced salt solution with 10 mM HEPES (StemCell Technologies) + 2% FBS + 0.05% NaN_3_. Anti-murine CD16/32 (clone 2.4G2, eBioscience) was used to block cells prior to antibody staining. Cells were stained with the following antibodies on ice for 30 min: CD45-APC, CD8α-FITC, CD3ε-PE, CD11b-e450 (eBioscience), F4/80-PE, SiglecF-TexasRed, FoxP3-V421, Ly6C-PerCP-Cy5.5 (BD Biosciences), Gr1-FITC, MHCII-V500, CD11c-BV605, CD25-APC, CD4-BV605, CD11b-APC, FasL-PE, CD19-PECy7, Ly6G-AF700, and NKp46-BV711 (Biolegend). Cells were fixed and permeabilized for 30 min using a transcription factor buffer set (eBioscience). For T regulatory cell identification, cells were stained with FoxP3-PECy7 or FoxP3-V421 for 1 h. All samples were acquired on a BD LSRFortessa (FACSDiva software, BD) and analyzed with FlowJo (TreeStar).

### Resazurin assay

The metabolic activity of 4T1 and MSC2 cells was measured using a colorimetric resazurin assay. Resazurin sodium salt (Sigma, Oakville, ON) was made up in 0.9% NaCl saline to a concentration of 4.4 μM. 1 × 10^4^ 4T1 or MSC2 cells were seeded in 24-well TC-treated plates and treated with gemcitabine at 10, 1, 0.5, 0.1, and 0.01 μM for 48 h. Physiological saline was used as a vehicle control. Resazurin was added to cells at a final concentration of 218 nM, and plates were read after 3–4 h by a 29 TECAN GENios plate reader using a 535-nm excitation and 590-nm emission filter. Experimental values are reported as normalized to cells grown in physiological saline.

### Statistical analyses

Student’s *t* tests with Welch’s correction were used for all comparisons using GraphPad Prism with *p < 0.05, **p < 0.01, and ***p < 0.001 indicating statistical significance. NS = data are not significantly different.

## Results

### CD11b^+^Gr1^+^ cells in the lungs of tumor-bearing mice

**Fig. 1:**
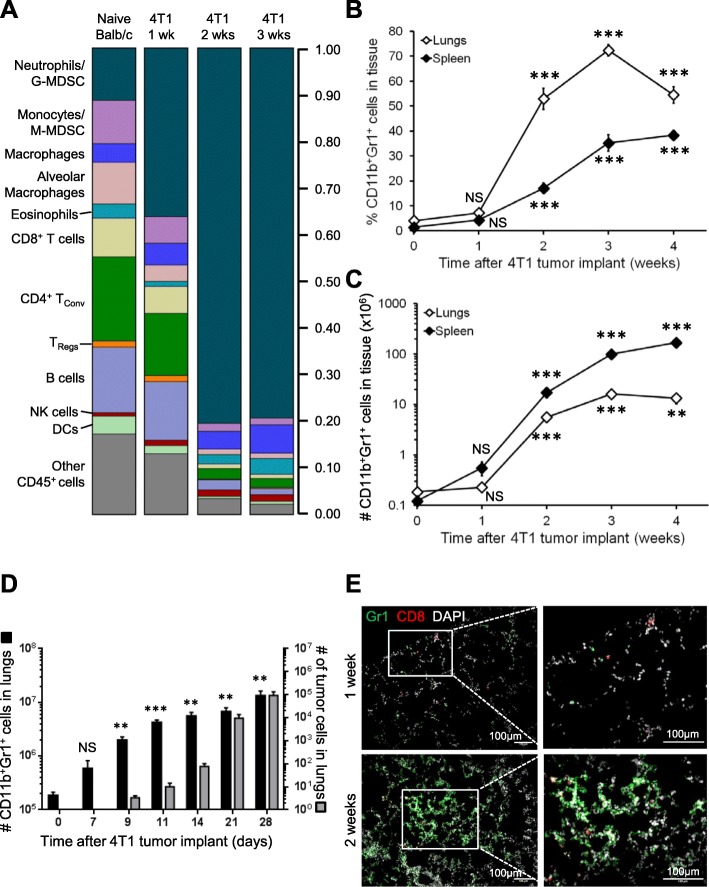
CD11b^+^Gr1^+^ cells accumulate in the lungs and spleens of 4T1 tumor-bearing mice. **a** Area plot illustrating mass cytometry time-of-flight (CyTOF) analysis of the proportion of various viable immune cell subsets recovered from the lungs of naïve mice or mice 1–3 weeks after orthotopic 4T1 mammary tumor implant. **b** Fraction of CD11b^+^Gr1^+^ cells recovered from the lungs and spleens of mice with 4T1 primary tumors. **c** Number of CD11b^+^Gr1^+^ cells in the lungs and spleens of mice with 4T1 primary tumors. Data are mean ± SEM with 5–15 mice per time point; significance compared to time 0. **d** Comparison between the numbers of CD11b^+^Gr1^+^ cells (left axis) and 4T1 tumor cells (right axis) in the lungs of 4T1 tumor-bearing mice with time after implant. Note different log scales on each axis; data are mean ± SEM with 4–6 mice per time point; significance compared to time 0. **e** Representative immunofluorescent images of lungs stained with Gr1 (green), CD8 (red), and DAPI (pseudo-colored white) from the lungs of mice 1 or 2 weeks after orthotopic 4T1 tumor implant. Increased magnification of boxed areas shown on the right. Scale bars = 100 μm

We used mass cytometry time-of-flight (CyTOF) to quantify immune cell subsets in the lungs of Balb/c mice bearing syngeneic, orthotopic 4T1 murine mammary tumors over time (Fig. 1a and Additional file 2: Figure S1). Consistent with previous reports [17–20], 4T1 tumors cause lung inflammation that primarily consists of CD11b^+^Gr1^+^ cells (i.e., neutrophils and/or G-MDSCs). We also found elevated proportions and total numbers of CD11b^+^ inflammatory macrophages, eosinophils, and NK cells in the lungs 3 weeks after 4T1 tumor implant relative to naïve tumor-free mice. The proportions of monocytes, alveolar macrophages, CD4^+^ conventional T cells (Tconvs), CD8^+^ T cells, and B cells in the lungs decreased by 2–3 weeks after tumor implant (Fig. 1a), but the absolute numbers of these cells did not change (Additional file 2: Figure S1). Growth kinetics for primary 4T1 tumors are shown in Additional file 3: Figure S2A. We used flow cytometry to validate the increased CD11b^+^Gr1^+^ cells in the lungs with time after 4T1 tumor implant (Fig. 1b, c) and also found an increased proportion and total number of CD11b^+^Gr1^+^ cells in the spleens of 4T1 tumor-bearing mice. The number of CD11b^+^Gr1^+^ cells increased up to 90-fold in the lungs and 1400-fold in the spleens by 3–4 weeks after tumor implantation (Fig. 1c). We also observed prominent splenomegaly in 4T1-bearing mice as primary tumor volume increased (Additional file 3: Figure S2B), consistent with previous reports [38]. We found a 42-fold increase in the number of proliferating cells in the spleens of 4T1 tumor-bearing mice (Additional file 3: Figure S2C), while the number of proliferating cells in the lungs did not increase. These data support extramedullary hematopoiesis in the spleens of 4T1 tumor-bearing mice [38] and indicate that the elevated CD11b^+^Gr1^+^ cell content in the lungs of these mice was not due to the proliferation of CD11b^+^Gr1^+^ cells within the lung tissue.

We were also interested in whether CD11b^+^Gr1^+^ cells arrive in the lungs prior to metastatic tumor cells and therefore compared CD11b^+^Gr1^+^ cells and 4T1 tumor cells in the lungs over time. We found that CD11b^+^Gr1^+^ cells were elevated within 7 days of primary tumor implant, while metastatic tumor cells were not detectable until 9 days after tumor implant (Fig. 1d). We used clonogenic assays to quantify metastatic tumor cell content in the lungs because this method is more sensitive than flow cytometry-based detection of fluorescently tagged tumor cells, and fluorescent tumor cells can induce a cytotoxic immune response in Balb/c mice [39–42]. Indeed, we were able to detect < 5 metastatic tumor cells in the lungs 9 days after primary tumor implant and 90 cells in the lungs 14 days after tumor implant (Fig. 1d; right-hand axis). Importantly, we observed aggregation of Gr1^+^ cells around CD8^+^ effector T cells in lung sections from mice 2 weeks after 4T1 tumor implant (Fig. 1e).

**Fig. 2:**
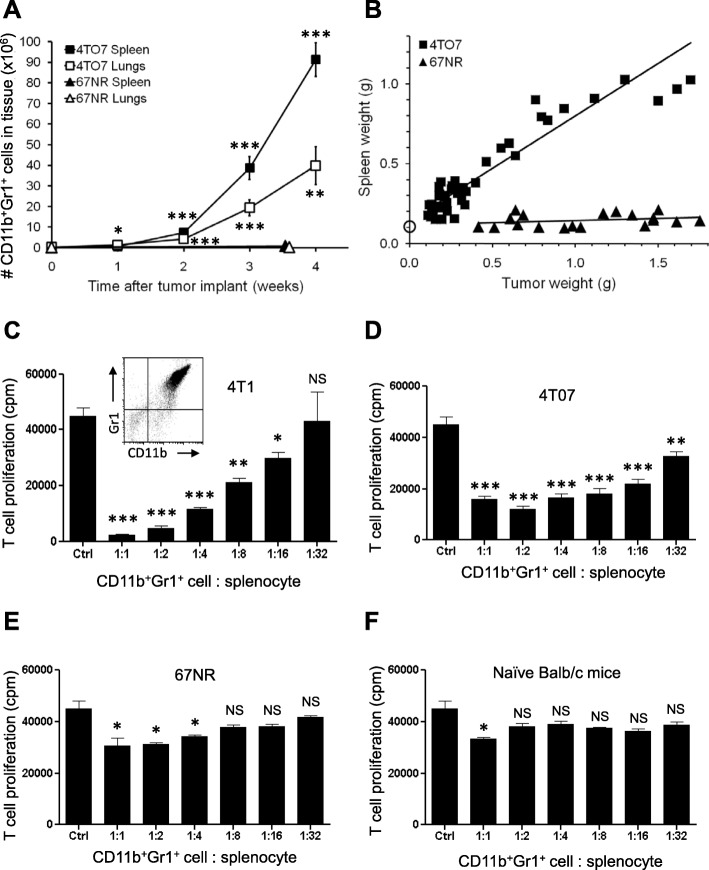
CD11b^+^Gr1^+^ cells from mice with 4T1 or 4T07 tumors, but not 67NR tumors, are immunosuppressive MDSCs*.* **a** Number of CD11b^+^Gr1^+^ cells in the lungs and spleens of mice with time after orthotopic 4T07 or 67NR mammary tumor implant. Data are mean ± SEM with 8–16 mice per time point (4T07) or 7–9 mice per time point (67NR); significance compared to time 0. **b** Tumor weight vs spleen weight for individual 4T07 or 67NR tumor-bearing mice. Open circle is spleen weight of *n* = 6 naïve mice. **c** CD11b^+^Gr1^+^ cells isolated from the lungs of 4T1 tumor-bearing mice suppress T cell proliferation in a concentration-dependent manner. ‘Ctrl’ bar is stimulated splenocytes alone. **d** CD11b^+^Gr1^+^ cells isolated from the lungs of 4T07 tumor-bearing mice suppress T cell proliferation in a concentration-dependent manner. **e** CD11b^+^Gr1^+^ cells isolated from the lungs of 67NR tumor-bearing mice only minimally suppress T cell proliferation. **f** CD11b^+^Gr1^+^ cells isolated from the lungs of naïve (tumor-free) mice are minimally suppressive. Data are mean ± SEM of triplicate wells; plots are representative of three independent experimental repeats. Significance compared to Ctrl

We next assessed whether other syngeneic murine mammary tumors induced the accumulation of CD11b^+^Gr1^+^ cells in the lungs. Similar to 4T1 tumors, metastatic 4T07 tumors induced accumulation of CD11b^+^Gr1^+^ cells in the lungs and spleen with time after orthotopic tumor implant (Fig. 2a), and we observed splenomegaly in 4T07-bearing mice (Fig. 2b). Conversely, non-metastatic 67NR tumors did not induce significant CD11b^+^Gr1^+^ cell accumulation in the lungs or spleen (Fig. 2a), and 67NR tumor-bearing mice did not exhibit splenomegaly (Fig. 2b), even in mice with large 67NR tumors. Using an antibody array, we observed elevated G-CSF levels in the serum of mice 3 weeks after 4T1 or 4T07 tumor implant (Additional file 4: Figure S3), but G-CSF was not detected in the circulation of naïve (tumor-free) mice or mice bearing 67NR tumors. Of note, primary tumors from all three cell lines were of comparable size (600–800 mg) 3 weeks after tumor implant. These data support a link between tumor-derived G-CSF expression and CD11b^+^Gr1^+^ cell expansion and accumulation in tissues [17].

### CD11b^+^Gr1^+^ cells in 4T1 and 4T07 tumor-bearing mice are MDSCs

CD11b and Gr1 are co-expressed on neutrophils and immune-suppressive MDSCs, and therefore, ex vivo functional assays are essential for the identification of MDSCs [24]. To determine whether the CD11b^+^Gr1^+^ cells in the lungs of naive and tumor-bearing mice are MDSCs, we used ex vivo assays to quantify the immune-suppressive function of these cells. We isolated Gr1^+^ cells from the lungs and spleens of 4T1 tumor-bearing mice and established that > 95% of the recovered Gr1^+^ cells co-expressed CD11b (Fig. 2c, inset). We found that CD11b^+^Gr1^+^ cells recovered from the lungs of 4T1 tumor-bearing mice suppressed the proliferation of activated T cells in a dose-dependent manner (Fig. 2c), indicating that these cells are indeed immunosuppressive MDSCs. Myeloid cells isolated from the lungs or spleens by Gr1 positive selection or Gr1 negative selection exhibited comparable immunosuppressive activities (Additional file 5: Figure S4A-B), and therefore, immunosuppressive function is not adversely affected by the Gr1 selection process. In addition to the spleen and lung, we found that CD11b^+^Gr1^+^ cells also increased in the liver, peripheral blood, bone marrow, and kidney 3 weeks after 4T1 tumor implant and that these CD11b^+^Gr1^+^ cells were immunosuppressive MDSCs (Additional file 5: Figure S4C-D). The kidney is not a metastatic target organ for 4T1 tumor cells, and these data provide evidence that MDSC accumulation is not restricted to metastatic tissues in this model.

As in 4T1 tumor-bearing mice, the CD11b^+^Gr1^+^ cells that accumulate in the lungs and spleens of mice with 4T07 tumors are also immunosuppressive MDSCs (Fig. 2d). In contrast, the small number of CD11b^+^Gr1^+^ cells present in the lungs and spleens of 67NR tumor-bearing mice exhibited low levels of immunosuppressive function (i.e., only at high CD11b^+^Gr1^+^ cell to splenocyte ratios; Fig. 2e). Similarly, CD11b^+^Gr1^+^ cells isolated from naïve (tumor-free) Balb/c mice were not immunosuppressive (Fig. 2f). Taken together, these data highlight the importance of using functional assays to identify CD11b^+^Gr1^+^ cells as MDSCs and demonstrate that both 4T1 and 4T07 tumors induce MDSC accumulation in the lungs.

### 4T1 tumor resection decreases serum G-CSF, but does not fully deplete MDSCs

**Fig. 3:**
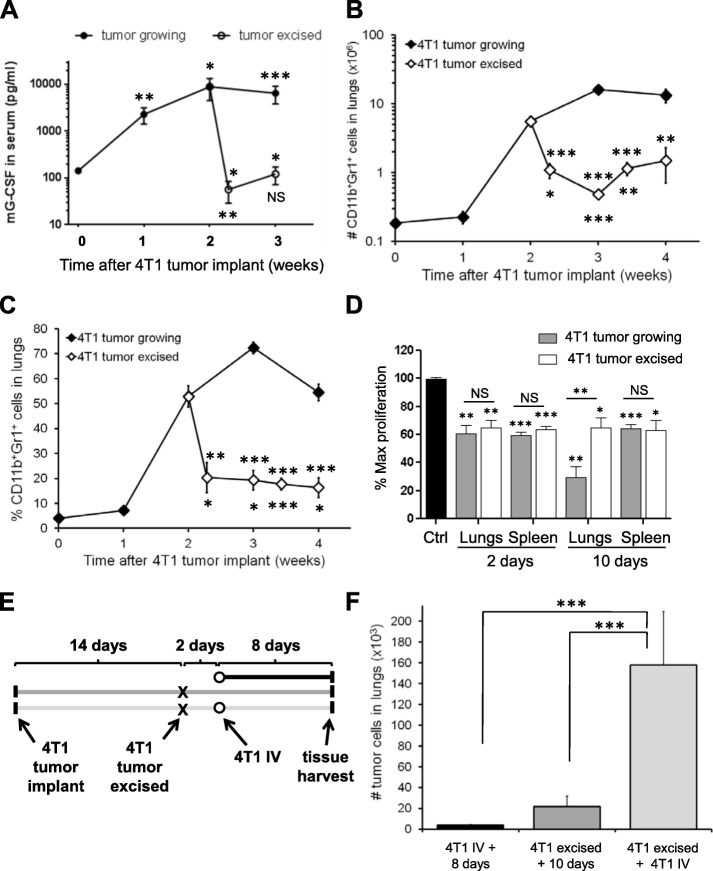
Functional MDSCs persist in the lungs after primary tumor resection and are associated with increased tumor growth in the lungs. **a** G-CSF in the peripheral blood of mice with 4T1 tumors or mice with 4T1 tumors resected 2 weeks after implantation. **b** Number of CD11b^+^Gr1^+^ cells in the lungs of mice with 4T1 primary tumors or mice with 4T1 tumors resected 2 weeks after implantation. **c** Proportion of CD11b^+^Gr1^+^ recovered from the lungs of mice with 4T1 primary tumors or mice with 4T1 tumors resected 2 weeks after implantation. Data are mean ± SEM with 4–8 mice per time point in the excised group. For the “tumor excised data”, stars above the curve indicate comparison to the unresected 2-week data point; stars below the curve indicate comparison to naïve mice. **d** CD11b^+^Gr1^+^ cells isolated from the spleens or lungs of mice 2 or 10 days after 4T1 tumor excision retain immunosuppressive function. Data are normalized to the fraction of stimulated T cell proliferation in the absence of CD11b^+^Gr1^+^ cells (Ctrl) and are mean ± SEM of two independent experimental repeats. Significance compared to Ctrl or as indicated. **e** Experimental outline for (**f**); mouse tissues were harvested 8 days after iv injection of 4T1 tumor cells, 10 days after resection of 4T1 primary tumors, or 8 days after iv injection of 4T1 tumor cells into mice with 4T1 primary tumors resected. **f** Total number of 4T1 tumor cells in the lungs of mice from (**e**). Data are mean ± SEM from 6 to 8 mice per group

We were interested in the longevity of MDSCs in the lungs after primary tumor resection, since the persistence of MDSCs in metastatic target organs may create an environment that promotes the secondary growth of disseminated tumor cells after surgery. We allowed 4T1 tumors to grow for 2 weeks to generate significant levels of MDSC accumulation in the lungs (Fig. 1b, c) with minimal metastatic disease (Fig. 1d) prior to surgical resection of the primary tumors. We found that serum G-CSF levels decreased to control levels within 48 h of tumor resection (Fig. 3a), indicating that primary 4T1 tumors are the main source of circulating G-CSF. Splenic MDSCs were significantly decreased within 2 days of primary tumor resection (Additional file 6: Figure S5A-B) concomitant with a reversion of splenomegaly (Additional file 6: Figure S5C) and a decrease in peripheral blood CD11b^+^Gr1^+^ cells (Additional file 6: Figure S5D). Interestingly, the number of CD11b^+^Gr1^+^ cells in the lungs decreased within 48 h of primary tumor resection, but did not return to control levels, remaining ~ 5.4-fold elevated relative to naïve mice for 2 weeks after tumor resection (Fig. 3b, c). CD11b^+^Gr1^+^ cells also remained elevated in the spleen and blood relative to naïve Balb/c mice for 2 weeks after tumor excision (Additional file 6: Figure S5). CD11b^+^Gr1^+^ cells that persisted in the spleen and lungs after primary tumor resection retained immunosuppressive function in the absence of the primary tumor (Fig. 3d) and were therefore MDSCs. These data indicate that significant numbers of functional MDSCs remain in the spleen and lungs 2 weeks after resection of 4T1 primary tumors.

### MDSCs increase 4T1 growth in the lungs after primary tumor resection

To assess the functional relevance of MDSCs that persist in the lungs after primary tumor resection, we allowed 4T1 tumors to grow for 2 weeks prior to surgical resection in order to “prime” the lungs with MDSCs. Two days after primary tumor resection, we iv injected 4T1 cells to directly seed the lungs and determine if persistent MDSCs influenced the survival and proliferation of these tumor cells in the lungs (Fig. 3e). We found ~ 21,600 spontaneously metastatic 4T1 tumor cells in the lungs 10 days after primary tumor excision and ~ 3900 4T1 tumor cells in the lungs of naïve mice 8 days after iv injection of 12,000 4T1 tumor cells. The presence of MDSCs in the lungs of mice 2 days after 4T1 tumor excision was associated with increased colonization of iv injected 4T1 tumor cells in the lungs by 40-fold (158,000 cells) relative to iv injected mice (Fig. 3f). IV injection of 4T1 tumor cells did not affect the number of CD11b^+^Gr1^+^ cells in the lungs (or spleens) of mice 8 days later, regardless of whether the iv injection was administered to a naïve mouse or a mouse 2 days after 4T1 tumor resection (Additional file 7: Figure S6), indicating that MDSC accumulation cannot be driven by a few thousand tumor cells in the lungs. Indeed, we have found that several million 4T1 tumor cells in the lungs are required to induce MDSC expansion and accumulation (data not shown). Taken together, these data demonstrate that immunosuppressive MDSCs persist in the lungs after surgical resection of primary tumors and are associated with profoundly increased growth of 4T1 tumor cells in the lungs.

### Targeting MDSCs with gemcitabine

**Fig. 4:**
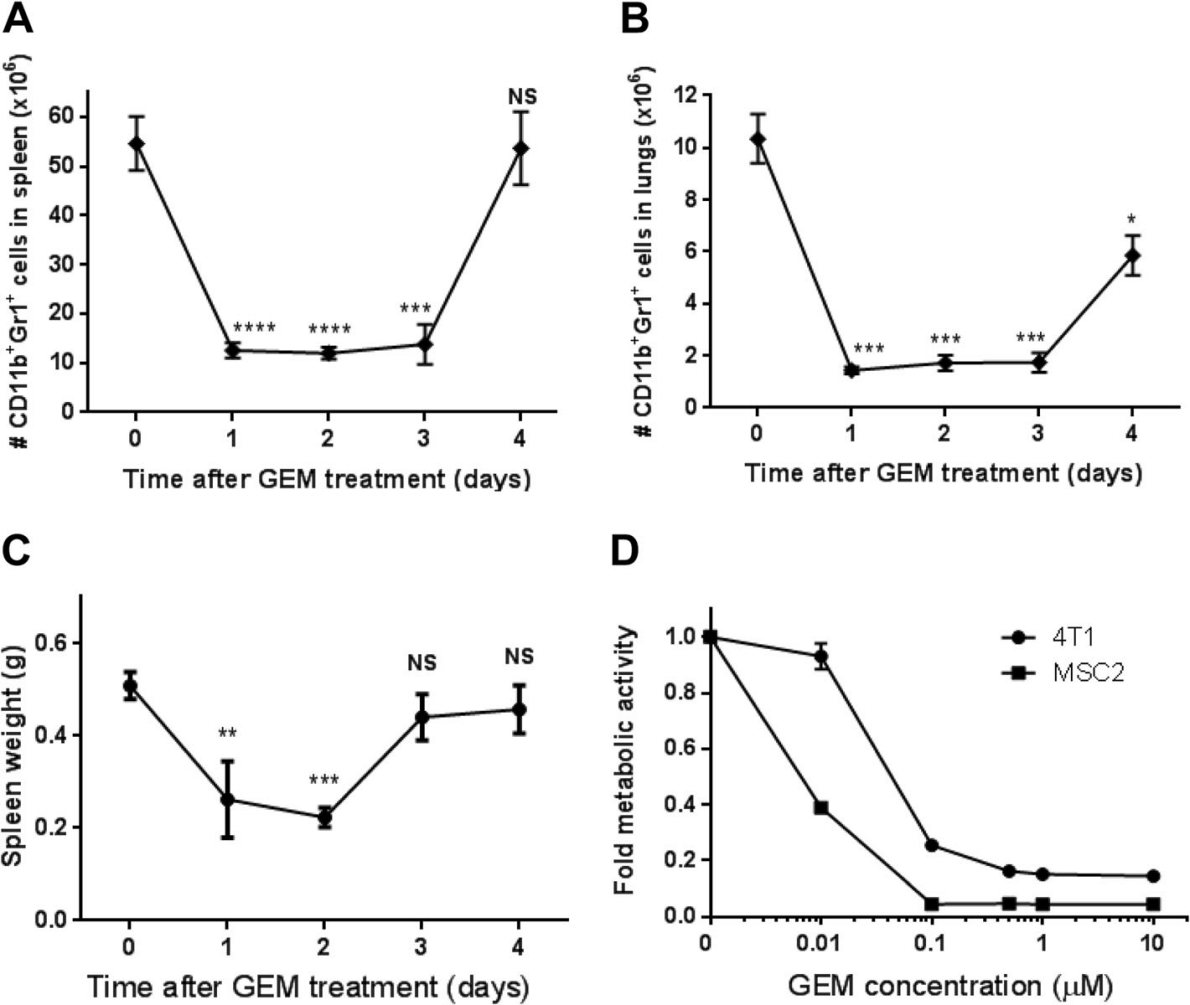
Gemcitabine depletes MDSCs in the spleen and lungs of 4T1 tumor-bearing mice. **a** Number of CD11b^+^Gr1^+^ cells in the spleens of 4T1 tumor-bearing mice after a single dose of 60 mg/kg gemcitabine administered 17 days after tumor implant. **b** Number of CD11b^+^Gr1^+^ cells in the lungs of 4T1 tumor-bearing mice after a single dose of 60 mg/kg gemcitabine administered 17 days after tumor implant. **c** Spleen weights of 4T1 tumor-bearing mice after gemcitabine treatment. Data are mean ± SEM from 4 to 6 mice per group. Significance compared to time 0. **d** Fold change in metabolic activity of 4T1 tumor cells and MSC2 myeloid cells treated with increasing doses of gemcitabine in vitro. Data are mean ± SEM from 6 experimental repeats; curves are significantly different (*p* < 0.001) as tested by ANOVA

We were interested in whether therapeutically targeting MDSCs that persist after surgery would decrease metastatic tumor growth in the lungs. Antibodies against G-CSF [17] or Gr1 [43, 44] have been shown to deplete MDSCs in different murine systems. Since G-CSF levels in the circulation were not associated with the presence of MDSCs after primary tumor resection (Fig. 3), we attempted to deplete MDSCs in the lungs of 4T1 tumor-bearing mice using anti-Gr1 antibody treatment. Contrary to previous reports in naïve or pathogen-stimulated mice [43, 44], anti-Gr1 treatment given by ip injection or intranasally failed to deplete MDSCs in the lungs of tumor-bearing mice (Additional file 8: Figure S7). The chemotherapeutic gemcitabine is used to treat a range of solid tumors and is preferentially cytotoxic to myeloid cells at low doses [45, 46]. To determine the magnitude and kinetics of gemcitabine toxicity toward MDSCs in the 4T1 model, mice were treated with a single dose of 60 mg/kg gemcitabine 17 days after primary tumor implantation. Within 24 h of gemcitabine treatment, MDSC levels were reduced by 77% in the spleen (Fig. 4a) and 84% in the lungs (Fig. 4b). MDSC levels and splenomegaly were decreased for 3–4 days in tumor-bearing mice (Fig. 4c) despite maintenance of large 4T1 primary tumors in these mice (Additional file 9: Figure S8A). To determine whether gemcitabine was inducing a cell cycle block in MDSCs, we analyzed the cell cycle profiles of whole splenocytes and lung cells from tumor-bearing mice after gemcitabine treatment. We observed a modest G1 block in spleen cells 24 h after gemcitabine treatment as indicated by increased G1 phase cells with concomitant decreases in S and G2 phase cells (Additional file 9: Figure S8B). We did not find gemcitabine-induced cell cycle profile changes at other time points in the spleen or lungs (Additional file 9: Figure S8), suggesting gemcitabine was not affecting MDSC levels by blocking proliferation. To determine the relative toxicity of gemcitabine toward 4T1 tumor cells or MDSCs, we utilized the immortalized MSC2 myeloid cell line [47] since the relatively poor ex vivo survival of MDSCs complicates the interpretation of drug toxicity studies. 4T1 tumor cells required an 8.2-fold higher dose of gemcitabine compared to MSC2 cells to produce a 60% reduction in metabolically active cells (Fig. 4d). Taken together, our data suggest the rapid decrease in MDSC levels observed in the spleen and lungs of 4T1 tumor-bearing mice after gemcitabine treatment was primarily due to gemcitabine-mediated cytotoxicity toward MDSCs.

### Targeting persistent MDSCs with low-dose gemcitabine decreases metastatic growth in the lungs

**Fig. 5:**
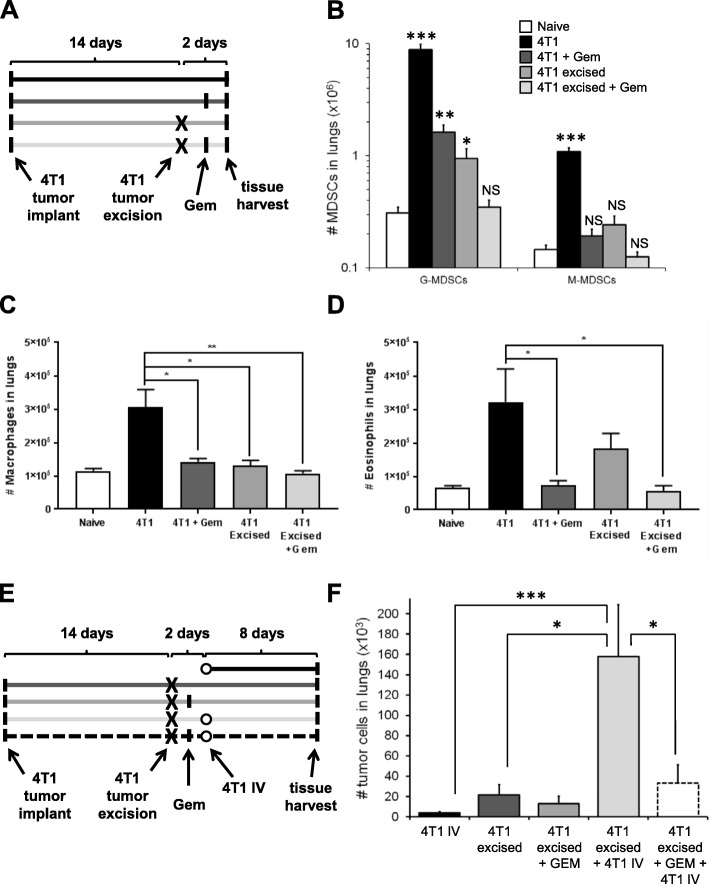
Gemcitabine decreases MDSCs that persist in the lungs after primary tumor resection and subsequent 4T1 growth in the lungs. **a** Experimental outline for (**b**–**d**); lung tissues were harvested as indicated from mice with or without 4T1 tumor resection and/or 60 mg/kg gemcitabine treatment for flow cytometry-based assessment of lung leukocytes. **b** Number of granulocytic-MDSCs or monocytic-MDSCs in the lungs of mice treated as in (**a**). Data are mean ± SEM of 7–8 mice per group. Significance compared to naïve (tumor-free) mice. **c** Number of macrophages in the lungs of mice treated as in (**a**). Data are mean ± SEM of 3–5 mice per group. **d** Number of eosinophils in the lungs of mice treated as in (**a**). Data are mean ± SEM of 3–5 mice per group. **e** Experimental outline for (**f**); lung tissues were harvested as indicated from mice with 4T1 tumor resection, gemcitabine treatment, and/or iv injection of 4T1 tumor cells prior to quantification of 4T1 cells in the lungs. **f** Total number of 4T1 tumor cells in the lungs of mice from (**e**). Data are mean ± SEM of 7–8 mice per group

We next used gemcitabine to target MDSCs that persist in the lungs following surgical resection of the primary tumor. As before, 4T1 tumors were allowed to grow for 2 weeks before surgical resection of the primary tumor. Mice were treated with a single dose of gemcitabine or PBS 1 day after tumor resection (or on day 15 for mice with 4T1 tumors still growing), and tissues were harvested the next day (Fig. 5a). Gemcitabine treatment of 4T1 tumor-bearing mice significantly reduced the levels of myeloid cells in the lungs 1 day later, including G-MDSCs, M-MDSCs (Fig. 5b), inflammatory macrophages (Fig. 5c), and eosinophils (Fig. 5d). Resection of primary 4T1 tumors significantly decreased G-MDSCs, M-MDSCs, and macrophages in the lungs (Fig. 5b, c), but did not significantly reduce eosinophils (Fig. 5d). Importantly, we did not observe changes in MDSCs, macrophages, eosinophils, or any other immune cell populations in the lungs of 4T1 tumor-bearing mice subjected to sham surgeries (Additional file 10: Figure S9). Neither gemcitabine treatment nor tumor resection significantly influenced alveolar macrophages, dendritic cells (DCs), NK cells, B cells, CD4^+^ T cells, or CD8^+^ T cells in the lungs (Additional file 11: Figure S10), which is consistent with previous reports that gemcitabine preferentially targets certain myeloid cell populations.

M-MDSCs are more immunosuppressive than G-MDSCs [22], and we previously reported that macrophages infiltrating the lungs of 4T1 tumor-bearing mice are potently immunosuppressive [35]. Importantly, the decrease of M-MDSCs and macrophages in the lungs after primary 4T1 tumor resection (Fig. 5b, c) indicates these cells are not directly involved in promoting 4T1 tumor growth in the lungs after primary tumor resection (Fig. 3f). Eosinophils remained elevated in the lungs after primary tumor resection (Fig. 5d), and to determine whether eosinophils influence 4T1 tumor growth in the lungs, we treated 4T1 tumor-bearing mice with an antibody against interleukin-5 (IL5) that has been shown to deplete eosinophils in murine model systems [48]. We found that depletion of eosinophils with anti-IL5 did not affect 4T1 metastatic growth in the lungs (Additional file 12: Figure S11), indicating that 4T1 tumor cells can grow in the lungs independent from eosinophil levels. Treating mice with gemcitabine after primary tumor resection reduced G-MDSCs in the lungs to control levels (Fig. 5b), eliminating the MDSCs that had persisted following surgery.

We then determined whether gemcitabine-mediated depletion of G-MDSCs that persist after primary tumor excision affected the growth of iv injected 4T1 tumor cells (Fig. 5e). Gemcitabine administered after primary tumor resection and before iv tumor cell injection significantly reduced the number of 4T1 tumor cells in the lungs (Fig. 5f). Taken together, these data indicate that the gemcitabine-mediated depletion of G-MDSCs that persist in the lungs after primary tumor resection decreases the development and growth of 4T1 tumor foci in the lungs.

## Discussion

A variety of bone marrow-derived cells have been implicated in supporting primary tumor growth and metastasis, and a better understanding of the expansion, phenotype, and longevity of these cells is required for the development of improved therapies to treat metastatic disease. Our findings indicate that, in addition to mice with 4T1 tumors, 4T07 tumor-bearing mice produce G-CSF and systemically induce the expansion of functional, immunosuppressive CD11b^+^Gr1^+^ MDSCs in the spleen and accumulation in the lungs. CD11b^+^Gr1^+^ cells isolated from mice implanted with non-metastatic 67NR tumors exhibited minimal T cell suppression at levels that were comparable to CD11b^+^Gr1^+^ cells from naïve mice. Profound proteomic differences have been reported between CD11b^+^Gr1^+^ cells isolated from the spleens of 4T1 or 67NR tumor-bearing mice [49], providing further evidence that CD11b^+^Gr1^+^ cells can be phenotypically and functionally distinct in mice with different mammary tumor types. The expansion and functional activation of CD11b^+^Gr1^+^ cells are influenced by tumor-derived factors [22], and recent work demonstrates that environmental factors can also induce the accumulation of CD11b^+^Gr1^+^ cells in tissues [50]. Low-grade chronic inflammation associated with obesity has been shown to drive the expansion of CD11b^+^Gr1^+^ cells within the lung in an IL-5 and GM-CSF-dependent manner, resulting in an increase in breast cancer pulmonary metastasis [50].

The lungs are a common site for breast cancer metastasis, and it is tempting to relate MDSC accumulation with pre-metastatic niche formation in the lungs. We found that MDSCs were detectable in the lungs prior to metastatic tumor cells, which is consistent with previously published pre-metastatic niche development kinetics [7, 8]. However, we found MDSCs accumulating systemically in metastatic and non-metastatic tissues (Additional file 5: Figure S4C-D), suggesting that immunosuppressive MDSCs are not specific to pre-metastatic niches or metastatic target organs in mice bearing 4T1 tumors. A similar phenotype was recently observed using the metastatic MMTV-polyoma middle T (PyMT) mammary tumor mouse model, where CD11b^+^Ly6G^+^ myeloid cells were mobilized to both metastatic and non-metastatic target organs [27]. In this model, accumulation of CD11b^+^Ly6G^+^ cells also occurred prior to tumor cell detection in the lungs and was abrogated in the absence of G-CSF.

Tumor-secreted factors, such as G-CSF, GM-CSF, TGF-β, and various interleukins, have been shown to drive the expansion of MDSCs, which then contribute to both an immunosuppressive tumor microenvironment and systemic dampening of the immune system [22]. Studies carried out in several mouse tumor models have shown that G-CSF is an important tumor-derived factor capable of altering myelopoiesis and inducing aberrant granulocytic MDSC expansion [17, 18, 38]. G-CSF loss- and gain-of-function approaches have shown that abrogating G-CSF production significantly diminishes MDSC accumulation in tissues, while over-expressing G-CSF or treatment of naïve mice with recombinant G-CSF induces MDSC accumulation [51]. We found that serum G-CSF levels decreased dramatically after surgical resection of primary 4T1 tumors, implicating the primary tumor as the main source of circulating G-CSF in these mice. Tumor resection also reduced MDSC levels in the spleen, peripheral blood, and lungs within 48 h. However, functional G-MDSCs remained significantly elevated relative to naïve mice for 2 weeks after tumor resection, indicating that continued production of G-CSF by the primary tumor is not required to maintain aberrantly high MDSC levels. Interestingly, the lungs of 4T1 tumor-bearing mice also contained elevated M-MDSCs, infiltrating macrophages, NK cells, and eosinophils (Fig. 1, Additional file 2: Figure S1), with M-MDSCs and macrophages returning to control levels 48 h after tumor resection (Fig. 5b–d). Eosinophils remained elevated in the lungs after primary tumor resection, which may be related to the high levels of IL-33 release we have previously observed in the lungs of 4T1 tumor-bearing mice [52] since IL-33 is known to activate eosinophils and induce eosinophilic airway inflammation [53]. Despite the increased eosinophil content in the lungs after tumor resection, we did not find that eosinophils affected 4T1 metastatic tumor growth in the lungs (Additional file 12: Figure S11).

One limitation of our study is that we were unable to extend our experimental timeline beyond 2 weeks post-tumor resection. We do not use radiation or chemotherapy after tumor resection, and due to the highly aggressive nature of the 4T1 tumor line, we observe regrowth of primary tumors in the surgical field 14 days after tumor resection. Whether this regrowth is due to tumor cells that were missed during the resection or due to metastasis of 4T1 cells from the lungs to the site of wound healing is an open question. Regardless, the propensity for tumor regrowth limits the timeframe of our experiments, and we are therefore unable to speculate on the longevity of suppressive lung MDSCs past the 2-week time point. Taken together, our data indicate that G-CSF-producing metastatic primary tumors create a pro-metastatic environment in the lungs consisting of several immunosuppressive myeloid cell types and that G-MDSCs persist in the lungs after primary tumor resection and are capable of promoting the growth of metastatic tumor foci.

Interestingly, the reduction in splenic MDSCs after tumor resection is consistent with some reports [31, 32], but depending on the surgical method, MDSC levels can increase in the spleen and bone marrow after 4T1 tumor resection when combined with abdominal nephrectomy [54, 55]. We did not observe a change in MDSCs or other immune cell types in the lungs of 4T1 tumor-bearing mice exposed to ‘sham’ surgery (i.e., without tumor resection; Additional file 10: Figure S9), confirming that the changes in the lung immune microenvironment found after tumor resection (Figs. 3 and 5) were due to removal of the tumor rather than the surgical procedure. With the metastasis promoting effects of MDSCs, it is important to determine whether MDSC populations persist in patients following surgical resection of primary breast tumors. Monitoring MDSC levels in the blood appears to be a suitable indication of MDSCs in tissues, and assessing circulating MDSCs in patients following surgical resection could identify patients at increased risk of developing metastatic disease. Indeed, elevated MDSC levels are observable in the peripheral blood of patients with metastatic cancer [56, 57], and increased MDSCs in the circulation of breast cancer patients correlates with clinical stage and decreased survival [58]. Circulating MDSC levels may be useful for screening and monitoring purposes, both before and after treatment, since breast cancer patients with elevated MDSCs may harbor immunosuppressive environments in peripheral tissues that could promote the development of secondary metastases after surgery.

Identifying therapeutic strategies that selectively target MDSCs could be used in patients after surgery to prevent subsequent metastatic growth. We found that a single dose of gemcitabine was sufficient to reduce G-MDSC, M-MDSCs, infiltrating macrophages, and eosinophils (Fig. 5b–d) in the lungs without affecting other myeloid or lymphoid cell types (Additional file 11: Figure S10). Gemcitabine-mediated depletion of G-MDSCs that persist in the lungs after primary tumor resection dramatically decreased tumor cell engraftment in an experimental model of lung metastasis. These data support the development of therapies that target G-MDSCs in concert with primary tumor removal for improved treatment of metastatic breast cancer. Clinical studies have demonstrated the efficacy of pharmacological strategies to reduce MDSC number (e.g., sunitinib) [59], to inhibit MDSC suppressive function (e.g., sildenafil) [60], or to differentiate MDSCs into mature myeloid cells (e.g., all-trans retinoic acid or 25-hydroxyvitamin D_3_) [28–30] in a variety of human cancers. We have previously shown that ATRA-mediated differentiation of MDSCs can promote metastatic tumor growth by generation of highly immunosuppressive macrophages [35], and therefore, strategies to target or inhibit MDSCs may produce more predictable outcomes. Directly targeting MDSCs with 5-fluorouracil [61] or gemcitabine [62] in various murine models of cancer significantly enhances T cell-dependent antitumor immunity, suggesting that therapeutics which target MDSCs may work synergistically with T cell-targeted therapies. Treatment with gemcitabine has been shown to deplete MDSCs in the peripheral blood of pancreatic cancer patients, as well as increase the ratio of T effector cells to T regulatory cells, indicating that targeting MDSCs can have additional downstream effects on immune cell populations critical for tumor rejection [63]. Combining cyclophosphamide or gemcitabine analogue treatment with adoptive dendritic cell therapy in the 4T1 breast carcinoma model was shown to increase activation of NKT cells, decrease tumor burden, and enhance protection against metastatic recurrence in the lungs [64]. Although treatment with cyclophosphamide or gemcitabine analogues decreased the frequency of MDSCs, these chemotherapeutics also promoted immunogenic cell death of 4T1 tumor cells and enhanced 4T1 immunogenicity by inducing the release and expression of immunogenic cell death-associated proteins [64]. Taken together, these studies suggest that targeting immunosuppressive cells in conjunction with immunotherapies that target T cells, such as anti-CTLA4 and anti-PD-L1, could be effective treatment strategies for tumor metastases.

## Conclusions

Our findings indicate that tumor-induced accumulation of MDSCs in the lungs can increase the growth of secondary metastatic tumors after resection of the primary tumor. Granulocytic MDSCs remain significantly elevated in the lungs of mice for 2 weeks after surgical resection of the primary tumor, creating an environment that promotes subsequent metastatic growth. Taken together, our data support the further development of strategies to monitor and therapeutically target MDSCs in breast cancer patients to reduce the development of tumor metastases.

## Additional files

Additional file 1: **Table S1.** Cell surface markers used to identify immune cell populations in the lungs by mass cytometry time-of-flight analysis in Fig 1a and Additional file 2: Figure S1A. (PDF 52 kb)

Additional file 2: **Figure S1.** CyTOF-based quantification of leukocyte populations in the lungs of naïve mice and mice 1, 2, or 3 weeks after orthotopic implantation of 4T1 murine mammary tumors. Data are mean ± SEM with *n* = 3 mice per group; *p < 0.05; **p < 0.01; ***p < 0.001; all other comparisons were not significantly different. (PDF 193 kb)

Additional file 3: **Figure S2.** A) Growth of 4T1 primary tumors after orthotopic implantation of 4T1 tumor cells. Data are mean ± SEM with *n* = 15–21 tumors per data point. B) Tumor weight vs spleen weight for individual 4T1 tumor-bearing mice. Open circle is average spleen weight of *n* = 6 naïve mice. C) Bromo-deoxyuridine (BrdU) labeled S phase cells in spleen and lungs of naïve mice or mice 3 weeks after 4T1 tumor implantation. Data are mean ± SEM with *n* = 4 mice per group. (PDF 290 kb)

Additional file 4: ** Figure S3.** Cytokine antibody array of plasma isolated from the peripheral blood of naïve mice or mice 3 weeks after implantation of 4T1, 4T07, or 67NR tumors. (PDF 321 kb)

Additional file 5: ** Figure S4.** A) % purity of CD11b^+^Gr1^+^ cells isolated from lungs or spleen of 4T1-bearing mice by Gr1 positive selection (+) or negative selection (−) with antibodies against CD4, CD5, CD11c, CD45R/B220, CD49b, CD117, TER119, and F4/80. Analysis gates set based on single stained control samples. B) CD11b^+^Gr1^+^ cells isolated by Gr1 positive selection are as immunosuppressive as CD11b^+^Gr1^+^ cells isolated by Gr1 negative selection, indicating the Gr1 antibody used in positive selection does not alter the immunosuppressive function of the cells. C**)** Accumulation of CD11b^+^Gr1^+^ cells in metastatic (lung, liver, bone marrow) and non-metastatic (spleen, peripheral blood, kidney) tissues 3 weeks after 4T1 tumor implant. Data are mean ± SEM with 4 mice per group. D) CD11b^+^Gr1^+^ cells isolated from tissues 3 weeks after 4T1 tumor implant suppress T cell proliferation. Data are mean ± SEM with 4 mice per group. Significance compared to stimulated splenocytes alone (RC). (PDF 123 kb)

Additional file 6: **Figure S5.** A) Number of CD11b^+^Gr1^+^ cells in the spleens of mice with 4T1 primary tumors or mice with 4T1 tumors resected 2 weeks after implantation. B) Proportion of CD11b^+^Gr1^+^ cells recovered from the spleens of mice with 4T1 primary tumors or mice with 4T1 tumors resected 2 weeks after implantation. C) Spleen weights of mice with 4T1 tumors or with 4T1 tumors surgically resected 2 weeks after implant. D) Proportion of CD45^+^ leukocytes that are CD11b^+^Gr1^+^ in the peripheral blood of mice with 4T1 tumors or with 4T1 tumors surgically resected 2 weeks after implant. Data are mean ± SEM with 4–8 mice per group. For the ‘tumor excised data’, stars above the curve indicate comparison to the unresected 2 week data point; stars below the curve indicate comparison to naïve mice. (PDF 101 kb)

Additional file 7: **Figure S6.** Intravenous injection of 12,000 4T1 tumor cells does not affect the number of CD11b^+^Gr1^+^ cells in the spleens or lungs of naïve mice or mice after 4T1 primary tumor resection. Data are mean ± SEM with *n* = 7–8 mice per group. (PDF 166 kb)

Additional file 8: ** Figure S7.** Treatment of 4T1-tumor-bearing mice with anti-Gr1 antibody does not deplete lung CD11b^+^Gr1^+^ cells. A) Representative flow plots of CD11b^+^Gr1^+^ cells in the lungs of 4T1-tumor bearing mice treated with 100 μg anti-Gr1 antibody or isotype control by intraperitoneal (IP) injection or by intranasal (IN) administration every 4 days until tissue harvest on day 21. B) Proportion of CD11b^+^Gr1^+^ cells recovered from the lungs of 4T1-tumor bearing mice treated with 100 μg anti-Gr1 antibody or isotype control. Data are mean ± SEM with *n* = 6 mice per group. (PDF 102 kb)

Additional file 9: **Figure S8.** A) 4T1 tumor weights after single injection of 60 mg/kg gemcitabine administered 17 days after primary tumor implant. B) Flow cytometry analysis of G1, S, and G2/M phase cells in the spleens of mice from (A). C) Flow cytometry analysis of G1, S, and G2/M phase cells in the lungs of mice from (A). Data are mean ± SEM with *n* = 5–6 mice per group. Significance compared to control (time 0) with *p < 0.05; ***p < 0.001; all other comparisons were not significantly different. (PDF 41 kb)

Additional file 10: **Figure S9.** Total numbers of CD11b^+^Gr1^+^ MDSCs, macrophages, alveolar macrophages, eosinophils, dendritic cells (DCs), B cells, CD8^+^ T cells, CD4^+^ T cells, and regulatory T cells (Tregs) in the lungs of 4T1 tumor-bearing mice and 4T1 tumor-bearing mice after sham surgery. None of the comparisons were significantly different. (PDF 122 kb)

Additional file 11: **Figure S10.** A) Total numbers of alveolar macrophages, dendritic cells (DCs), B cells, NK cells, CD8^+^ T cells, CD4^+^ T cells, and regulatory T cells (Tregs) in the lungs of mice from the experiment outlined in Fig. 5. None of the comparisons were significantly different. (PDF 99 kb)

Additional file 12: **Figure S11.** A) Numbers of eosinophils in the lungs of 4T1 tumor-bearing mice treated with anti-IL5 antibody (clone TRFK5) or isotype control. B) Numbers of 4T1 tumor cells in the lungs of 4T1 tumor-bearing mice treated with anti-IL5 antibody or isotype control. Data are mean ± SEM with *n* = 4–10 mice per group. (PDF 241 kb)

## Data Availability

Not applicable.
