# Supplementary material for: Targeting myeloid-derived suppressor cells in combination with primary mammary tumor resection reduces metastatic growth in the lungs
Source: Breast Cancer Res. 2019 Sep 5;21:103. doi: 10.1186/s13058-019-1189-x (PMC6727565; doi:10.1186/s13058-019-1189-x)
Supplement: Supplementary file 1 — Table S1. Cell surface markers used to identify immune cell populations in the lungs by mass cytometry time-of-flight analysis in Fig 1a and Additional file 2: Figure S1A. (PDF 52 kb) [file 13058_2019_1189_MOESM1_ESM.pdf]

Supplemental Table 1

SUBSET	MARKERS	SUBSET	MARKERS	SUBSET	MARKERS	SUBSET	MARKERS
**G-MDSC/ Neutrophils**	$NK1.1^-$<br>$CD19^-$<br>$CD11b^+$<br>$SiglecF^-$<br>$Ly6G^+$	**Alveolar MΦ**	$NK1.1^-$<br>$CD19^-$<br>$CD11b^-$<br>$SiglecF^+$	**$CD8^+$ T cells**	$NK1.1^-$<br>$CD19^-$<br>$CD11b^-$<br>$SiglecF^-$<br>$CD11c^-$<br>$CD8^+$	**B cells**	$CD19^+$<br>$NK1.1^-$
**M-MDSC/ Monocytes**	$NK1.1^-$<br>$CD19^-$<br>$CD11b^+$<br>$SiglecF^-$<br>$Ly6G^-$<br>$Ly6C^{-/mid}$<br>$MHCII^-$	**Eosinophils**	$NK1.1^-$<br>$CD19^-$<br>$CD11b^+$<br>$SiglecF^+$	**$CD4^+$ T_{conv} cells**	$NK1.1^-$<br>$CD19^-$<br>$CD11b^-$<br>$SiglecF^-$<br>$CD11c^-$<br>$CD4^+$<br>$CD25^-$	**NK cells**	$NK1.1^+$<br>$CD19^-$
**Macrophages (MΦ)**	$NK1.1^-$<br>$CD19^-$<br>$CD11b^+$<br>$SiglecF^-$<br>$Ly6G^-$<br>$Ly6C^+$	**Dendritic cells (DCs)**	$NK1.1^-$<br>$CD19^-$<br>$SiglecF^-$<br>$CD11c^+$<br>$MHCII^{-/+}$	**T_{regs}**	$NK1.1^-$<br>$CD19^-$<br>$CD11b^-$<br>$SiglecF^-$<br>$CD11c^-$<br>$CD4^+$<br>$CD25^+$		

Supplemental Table 1: Cell surface markers used to identify immune cell populations for CyTOF analysis in figure 1A and supplemental figure 1A.
