# Supplementary material for: Targeting myeloid-derived suppressor cells in combination with primary mammary tumor resection reduces metastatic growth in the lungs
Source: Breast Cancer Res. 2019 Sep 5;21:103. doi: 10.1186/s13058-019-1189-x (PMC6727565; doi:10.1186/s13058-019-1189-x)
Supplement: Supplementary file 2 — Figure S1. CyTOF-based quantification of leukocyte populations in the lungs of naïve mice and mice 1, 2, or 3 weeks after orthotopic implantation of 4T1 murine mammary tumors. Data are mean ± SEM with n = 3 mice per group; *p < 0.05; **p < 0.01; ***p < 0.001; all other comparisons were not significantly different. (PDF 193 kb) [file 13058_2019_1189_MOESM2_ESM.pdf]

Supplemental Figure 1

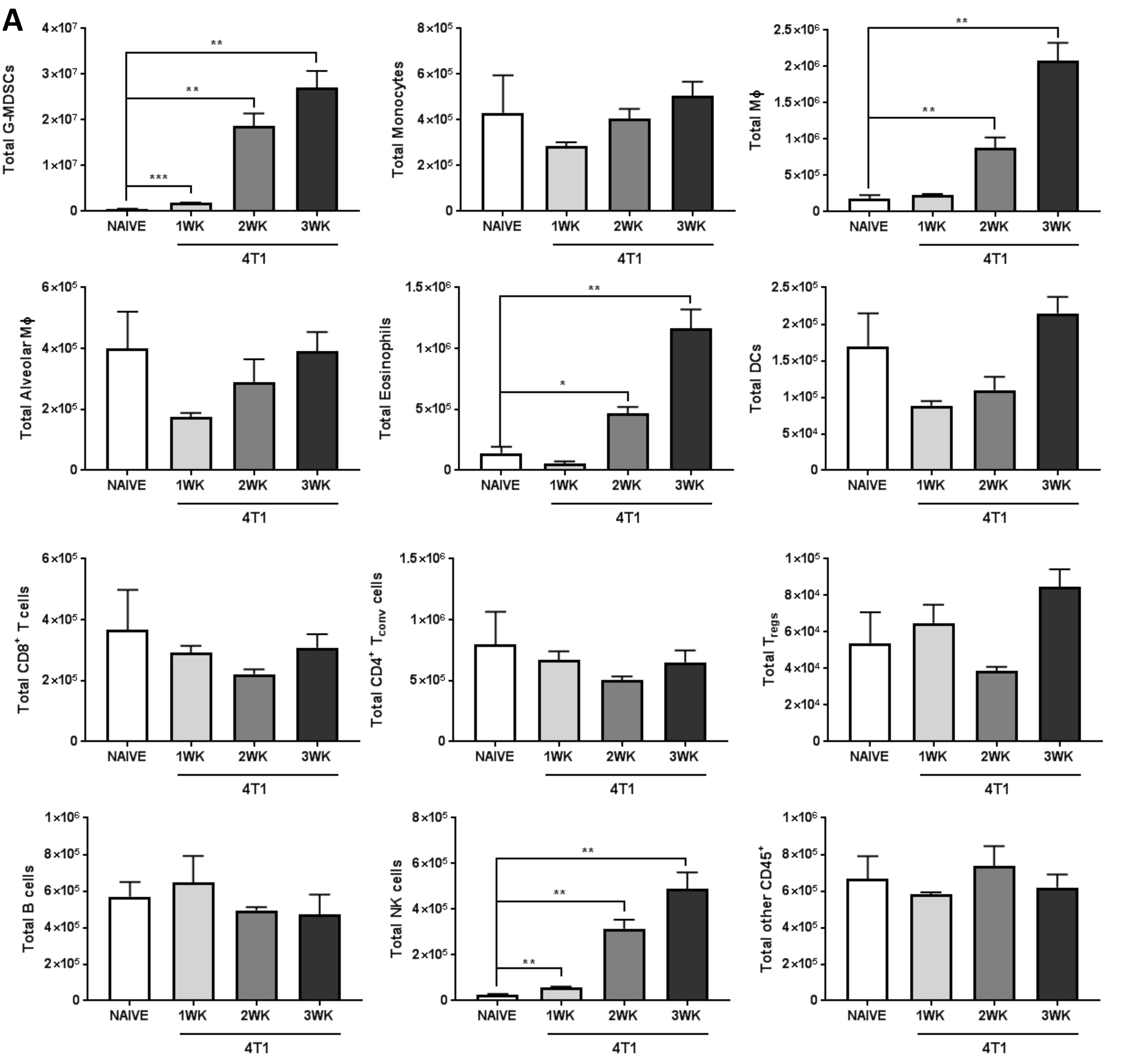

Supplemental Figure 1: **A)** Quantification of lung-infiltrating leukocyte populations by CyTOF analysis in naïve mice and mice implanted with 4T1 orthotopic mammary tumors 1 week, 2 weeks, and 3 weeks post-implant. Data are mean ± SEM with n=3 mice per group; *p<0.05; **p<0.01; ***p<0.001.
