# Supplementary material for: Targeting myeloid-derived suppressor cells in combination with primary mammary tumor resection reduces metastatic growth in the lungs
Source: Breast Cancer Res. 2019 Sep 5;21:103. doi: 10.1186/s13058-019-1189-x (PMC6727565; doi:10.1186/s13058-019-1189-x)
Supplement: Supplementary file 3 — Figure S2. A) Growth of 4T1 primary tumors after orthotopic implantation of 4T1 tumor cells. Data are mean ± SEM with n = 15–21 tumors per data point. B) Tumor weight vs spleen weight for individual 4T1 tumor-bearing mice. Open circle is average spleen weight of n = 6 naïve mice. C) Bromo-deoxyuridine (BrdU) labeled S phase cells in spleen and lungs of naïve mice or mice 3 weeks after 4T1 tumor implantation. Data are mean ± SEM with n = 4 mice per group. (PDF 290 kb) [file 13058_2019_1189_MOESM3_ESM.pdf]

Supplemental Figure 2

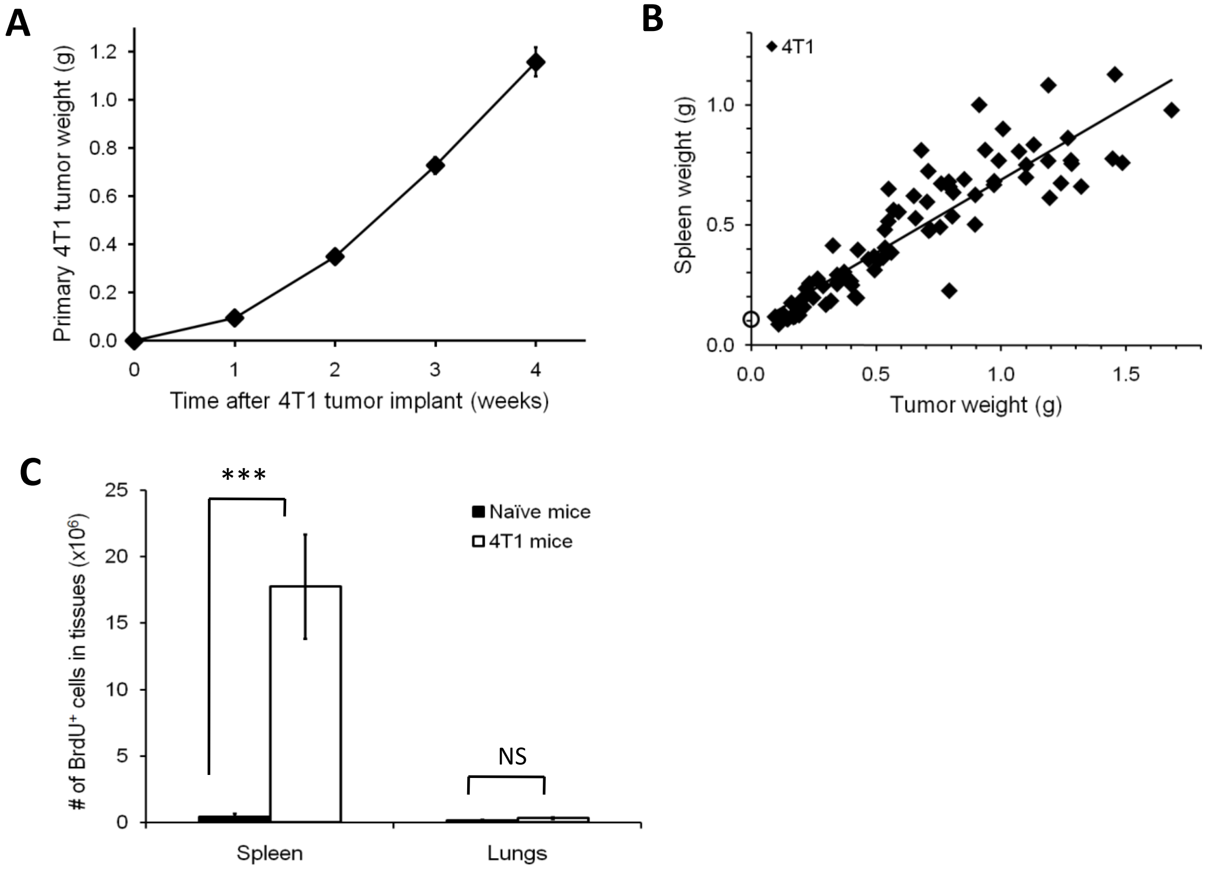

Supplemental Figure 2: **A)** Growth of 4T1 primary tumors after orthotopic implantation of 4T1 tumor cells. Data are mean ± sem with n=15-21 tumors per data point. **B)** Tumor weight vs spleen weight for individual 4T1 tumor-bearing mice. Open circle is spleen weight of n=6 naïve mice **C)** Bromo-deoxyuridine (BrdU) labeled S phase cells in spleen and lungs of naïve mice or mice 3 weeks after 4T1 tumour implantation. Data are mean ± sem with n=4 tumors per group; ***p<0.001
