# Supplementary material for: Targeting myeloid-derived suppressor cells in combination with primary mammary tumor resection reduces metastatic growth in the lungs
Source: Breast Cancer Res. 2019 Sep 5;21:103. doi: 10.1186/s13058-019-1189-x (PMC6727565; doi:10.1186/s13058-019-1189-x)
Supplement: Supplementary file 4 — Figure S3. Cytokine antibody array of plasma isolated from the peripheral blood of naïve mice or mice 3 weeks after implantation of 4T1, 4T07, or 67NR tumors. (PDF 321 kb) [file 13058_2019_1189_MOESM4_ESM.pdf]

Supplemental Figure 3

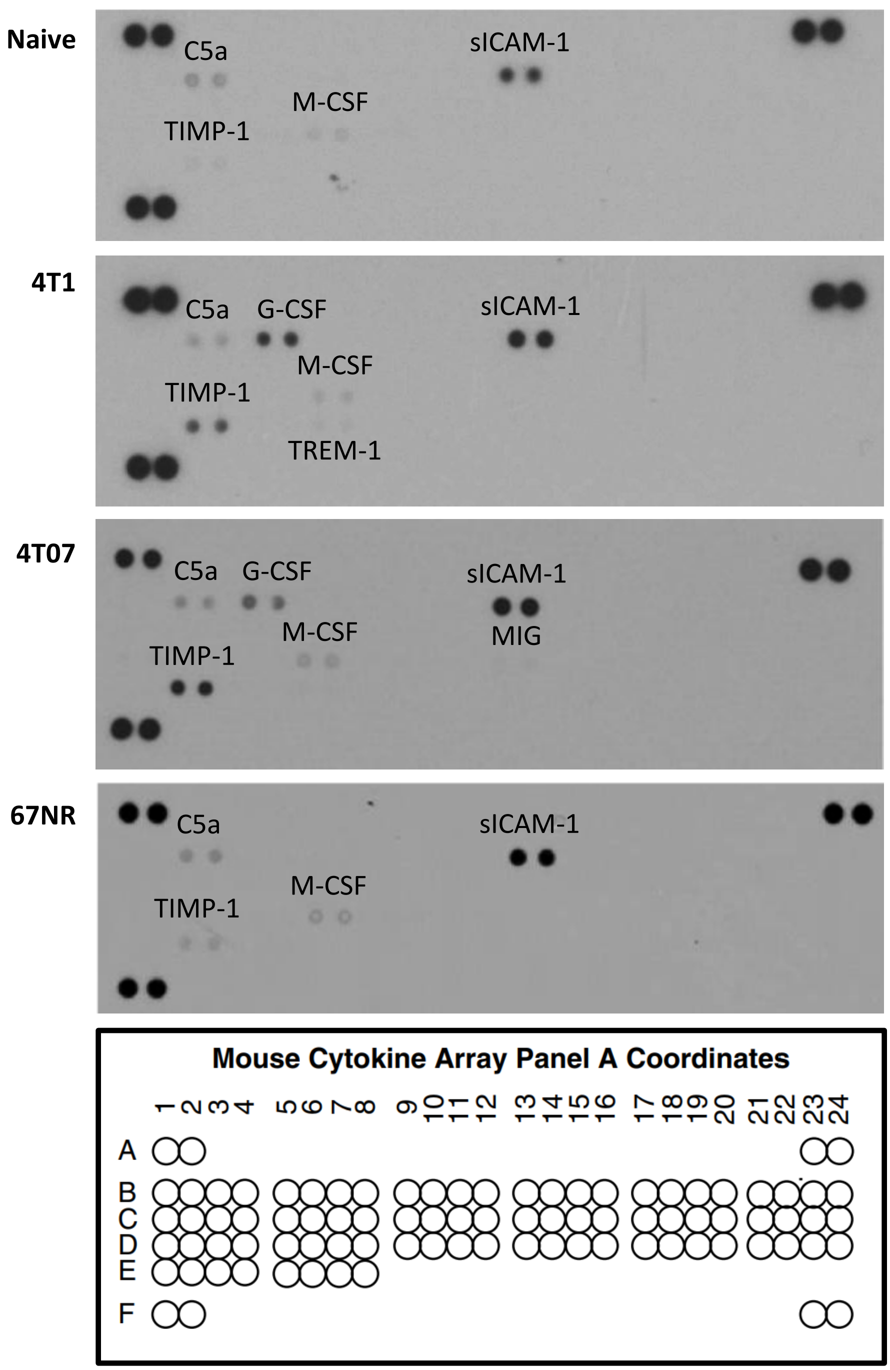

Coordinate	Target/Control
A1, A2	Reference Spot
A23, A24	Reference Spot
B1, B2	BLC
B3, B4	C5/C5a
B5, B6	G-CSF
B7, B8	GM-CSF
B9, B10	I-309
B11, B12	Eotaxin
B13, B14	sICAM-1
B15, B16	IFN-γ
B17, B18	IL-1α
B19, B20	IL-1β
B21, B22	IL-1ra
B23, B24	IL-2
C1, C2	IL-3
C3, C4	IL-4
C5, C6	IL-5
C7, C8	IL-6
C9, C10	IL-7
C11, C12	IL-10
C13, C14	IL-13
C15, C16	IL-12 p70
C17, C18	IL-16
C19, C20	IL-17
C21, C22	IL-23
C23, C24	IL-27
D1, D2	IP-10
D3, D4	I-TAC
D5, D6	KC
D7, D8	M-CSF
D9, D10	JE
D11, D12	MCP-5
D13, D14	MIG
D15, D16	MIP-1α
D17, D18	MIP-1β
D19, D20	MIP-2
D21, D22	RANTES
D23, D24	SDF-1
E1, E2	TARC
E3, E4	TIMP-1
E5, E6	TNF-α
E7, E8	TREM-1
F1, F2	Reference Spot
F23, F24	PBS (Negative Control)

Supplemental Figure 3: Cytokine antibody array of plasma isolated from the peripheral blood of naïve mice or mice 3 weeks after implant of 4T1, 4T07, or 67NR tumours.
