# Supplementary material for: Targeting myeloid-derived suppressor cells in combination with primary mammary tumor resection reduces metastatic growth in the lungs
Source: Breast Cancer Res. 2019 Sep 5;21:103. doi: 10.1186/s13058-019-1189-x (PMC6727565; doi:10.1186/s13058-019-1189-x)
Supplement: Supplementary file 5 — Figure S4. A) % purity of CD11b+Gr1+ cells isolated from lungs or spleen of 4T1-bearing mice by Gr1 positive selection (+) or negative selection (−) with antibodies against CD4, CD5, CD11c, CD45R/B220, CD49b, CD117, TER119, and F4/80. Analysis gates set based on single stained control samples. B) CD11b+Gr1+ cells isolated by Gr1 positive selection are as immunosuppressive as CD11b+Gr1+ cells isolated by Gr1 negative selection, indicating the Gr1 antibody used in positive selection does not alter the immunosuppressive function of the cells. C) Accumulation of CD11b+Gr1+ cells in metastatic (lung, liver, bone marrow) and non-metastatic (spleen, peripheral blood, kidney) tissues 3 weeks after 4T1 tumor implant. Data are mean ± SEM with 4 mice per group. D) CD11b+Gr1+ cells isolated from tissues 3 weeks after 4T1 tumor implant suppress T cell proliferation. Data are mean ± SEM with 4 mice per group. Significance compared to stimulated splenocytes alone (RC). (PDF 123 kb) [file 13058_2019_1189_MOESM5_ESM.pdf]

Supplemental Figure 4

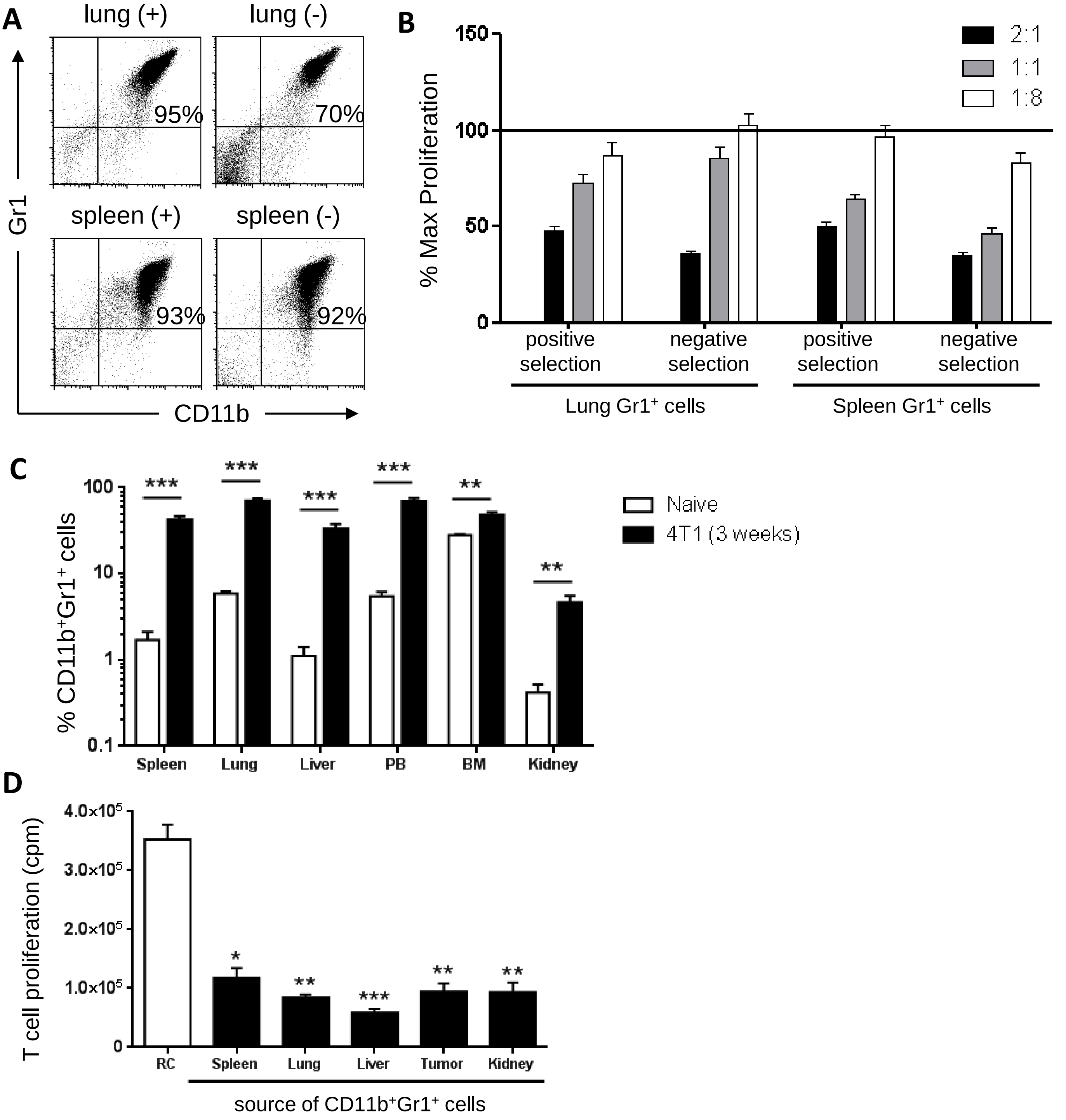

Supplemental Figure 4: **A)** % purity of $CD11b^+Gr1^+$ cells isolated from lungs or spleen of 4T1-bearing mice by Gr1 positive selection (+) or negative selection (-) with antibodies against CD4, CD5, CD11c, CD45R/B220, CD49b, CD117, TER119, and F4/80. Analysis gates set based on single stained control samples. **B)** $CD11b^+Gr1^+$ cells isolated by Gr1 positive selection are as immune suppressive as $CD11b^+Gr1^+$ cells isolated by Gr1 negative selection, indicating the Gr1 antibody used in positive selection does not alter the immune suppressive function of the cells. **C)** Accumulation of $CD11b^+Gr1^+$ cells in metastatic (lung, liver) and non-metastatic (spleen, peripheral blood, bone marrow, kidney) tissues 3 weeks post-4T1 implant. **D)** $CD11b^+Gr1^+$ cells isolated from tissues 3 weeks after 4T1 primary tumor implant suppress T cell proliferation.
