# Supplementary material for: Targeting myeloid-derived suppressor cells in combination with primary mammary tumor resection reduces metastatic growth in the lungs
Source: Breast Cancer Res. 2019 Sep 5;21:103. doi: 10.1186/s13058-019-1189-x (PMC6727565; doi:10.1186/s13058-019-1189-x)
Supplement: Supplementary file 6 — Figure S5. A) Number of CD11b+Gr1+ cells in the spleens of mice with 4T1 primary tumors or mice with 4T1 tumors resected 2 weeks after implantation. B) Proportion of CD11b+Gr1+ cells recovered from the spleens of mice with 4T1 primary tumors or mice with 4T1 tumors resected 2 weeks after implantation. C) Spleen weights of mice with 4T1 tumors or with 4T1 tumors surgically resected 2 weeks after implant. D) Proportion of CD45+ leukocytes that are CD11b+Gr1+ in the peripheral blood of mice with 4T1 tumors or with 4T1 tumors surgically resected 2 weeks after implant. Data are mean ± SEM with 4–8 mice per group. For the ‘tumor excised data’, stars above the curve indicate comparison to the unresected 2 week data point; stars below the curve indicate comparison to naïve mice. (PDF 101 kb) [file 13058_2019_1189_MOESM6_ESM.pdf]

Supplemental Figure 5

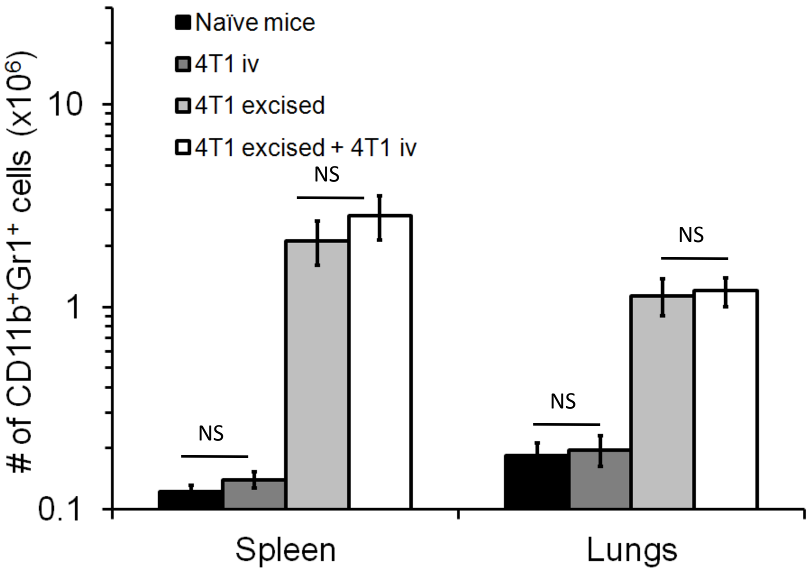

Supplemental Figure 6: Intravenous injection of 12,000 4T1 tumor cells does not affect the number of CD11b+Gr1+ cells in the spleen or lungs of naïve mice or mice after 4T1 primary tumour resection.
