# Supplementary material for: Targeting myeloid-derived suppressor cells in combination with primary mammary tumor resection reduces metastatic growth in the lungs
Source: Breast Cancer Res. 2019 Sep 5;21:103. doi: 10.1186/s13058-019-1189-x (PMC6727565; doi:10.1186/s13058-019-1189-x)
Supplement: Supplementary file 7 — Figure S6. Intravenous injection of 12,000 4T1 tumor cells does not affect the number of CD11b+Gr1+ cells in the spleens or lungs of naïve mice or mice after 4T1 primary tumor resection. Data are mean ± SEM with n = 7–8 mice per group. (PDF 166 kb) [file 13058_2019_1189_MOESM7_ESM.pdf]

Supplemental Figure 6

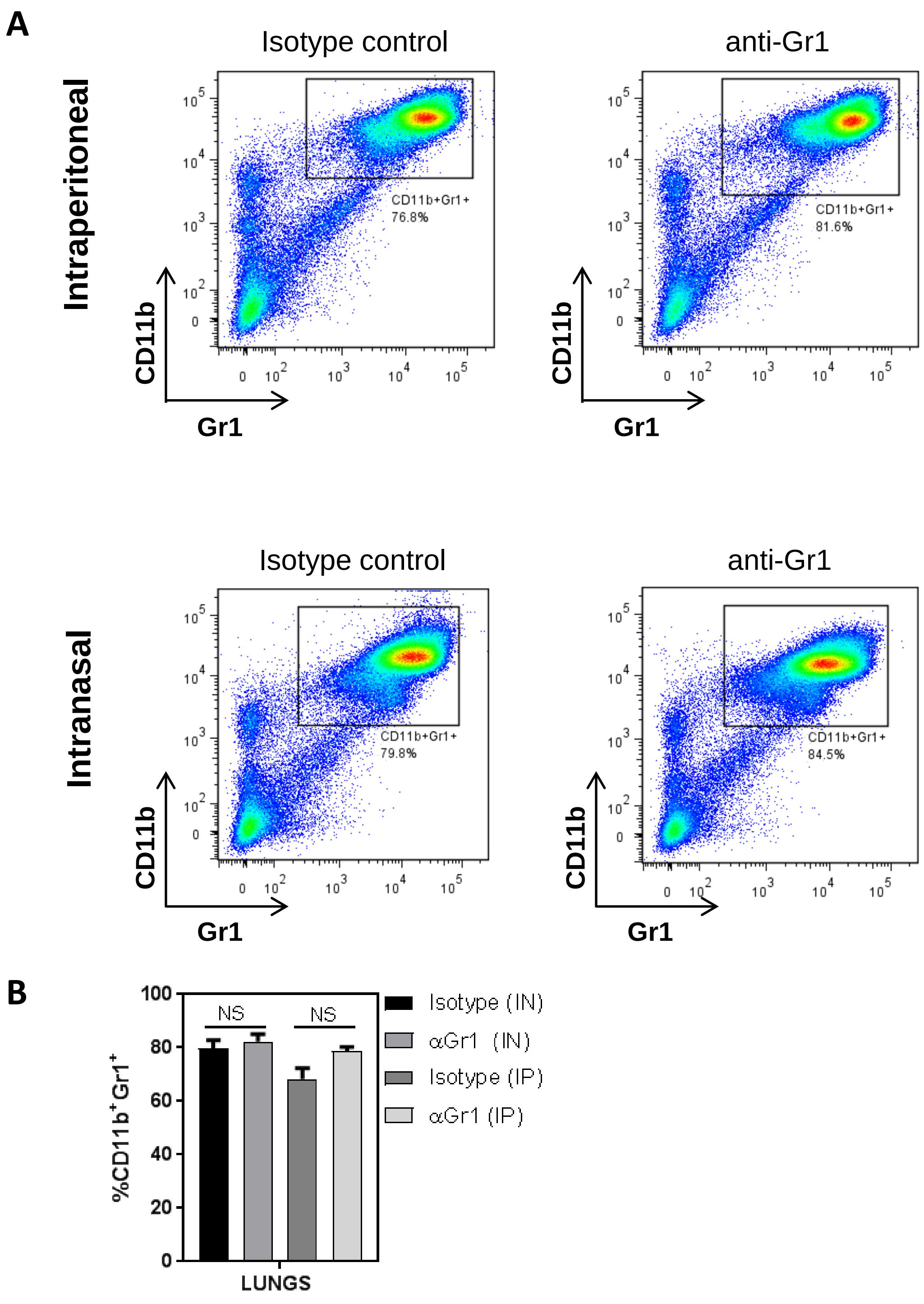

Supplemental Figure 7: Treatment of 4T1-tumour-bearing mice treated with anti-Gr1 antibody did not deplete lung CD11b$^+$Gr1$^+$ cells. **A)** Representative flow plots of %CD11b$^+$Gr1$^+$ cells in the lungs of 4T1-tumor bearing mice treated with 100ug anti-Gr1 antibody or isotype control (intraperitoneal injection or intranasally) every 4 days until tissue harvest on day 21. **B)** Quantification of %CD11b$^+$Gr1$^+$ cells in the lungs of 4T1-tumor bearing mice treated with 100ug anti-Gr1 antibody or isotype control. n=6 mice per group.
