# Supplementary material for: Targeting myeloid-derived suppressor cells in combination with primary mammary tumor resection reduces metastatic growth in the lungs
Source: Breast Cancer Res. 2019 Sep 5;21:103. doi: 10.1186/s13058-019-1189-x (PMC6727565; doi:10.1186/s13058-019-1189-x)
Supplement: Supplementary file 8 — Figure S7. Treatment of 4T1-tumor-bearing mice with anti-Gr1 antibody does not deplete lung CD11b+Gr1+ cells. A) Representative flow plots of CD11b+Gr1+ cells in the lungs of 4T1-tumor bearing mice treated with 100 μg anti-Gr1 antibody or isotype control by intraperitoneal (IP) injection or by intranasal (IN) administration every 4 days until tissue harvest on day 21. B) Proportion of CD11b+Gr1+ cells recovered from the lungs of 4T1-tumor bearing mice treated with 100 μg anti-Gr1 antibody or isotype control. Data are mean ± SEM with n = 6 mice per group. (PDF 102 kb) [file 13058_2019_1189_MOESM8_ESM.pdf]

Supplemental Figure 7

A

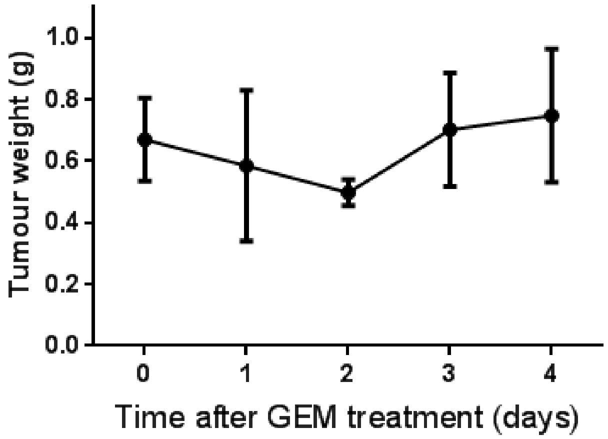

B

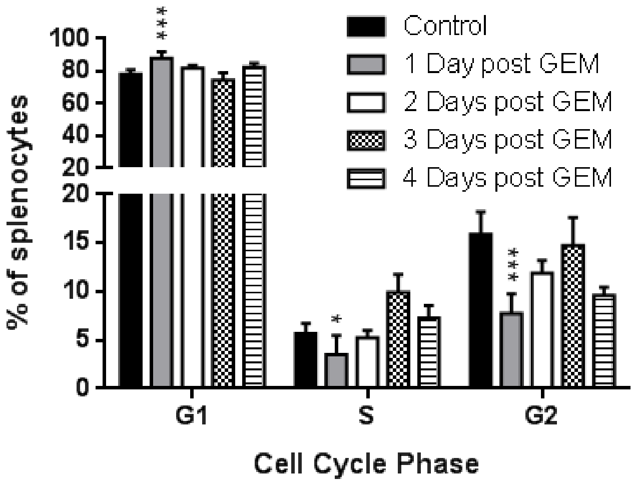

C

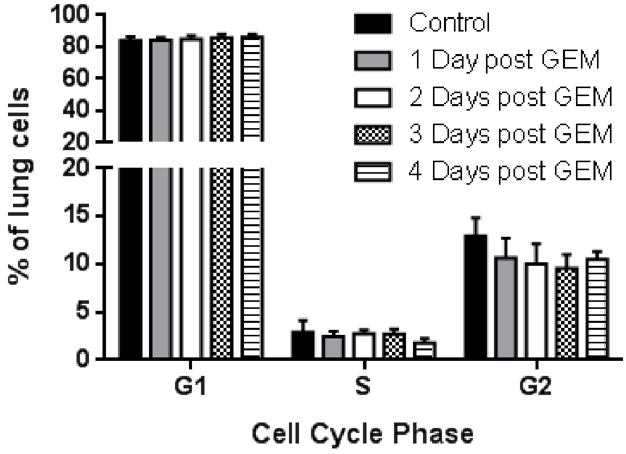

Supplemental Figure 8: **A)** 4T1 tumour weights after single injection of 60mg/kg gemcitabine administered 17 days after primary tumour implant. **B)** Flow cytometry analysis of G1, S, and G2/M phase cells in the spleen from mice in (A). **C)** Flow cytometry analysis of G1, S, and G2/M phase cells in the lungs from mice in (A).
