# Supplementary material for: Targeting myeloid-derived suppressor cells in combination with primary mammary tumor resection reduces metastatic growth in the lungs
Source: Breast Cancer Res. 2019 Sep 5;21:103. doi: 10.1186/s13058-019-1189-x (PMC6727565; doi:10.1186/s13058-019-1189-x)
Supplement: Supplementary file 9 — Figure S8. A) 4T1 tumor weights after single injection of 60 mg/kg gemcitabine administered 17 days after primary tumor implant. B) Flow cytometry analysis of G1, S, and G2/M phase cells in the spleens of mice from (A). C) Flow cytometry analysis of G1, S, and G2/M phase cells in the lungs of mice from (A). Data are mean ± SEM with n = 5–6 mice per group. Significance compared to control (time 0) with *p < 0.05; ***p < 0.001; all other comparisons were not significantly different. (PDF 41 kb) [file 13058_2019_1189_MOESM9_ESM.pdf]

Supplemental Figure 8

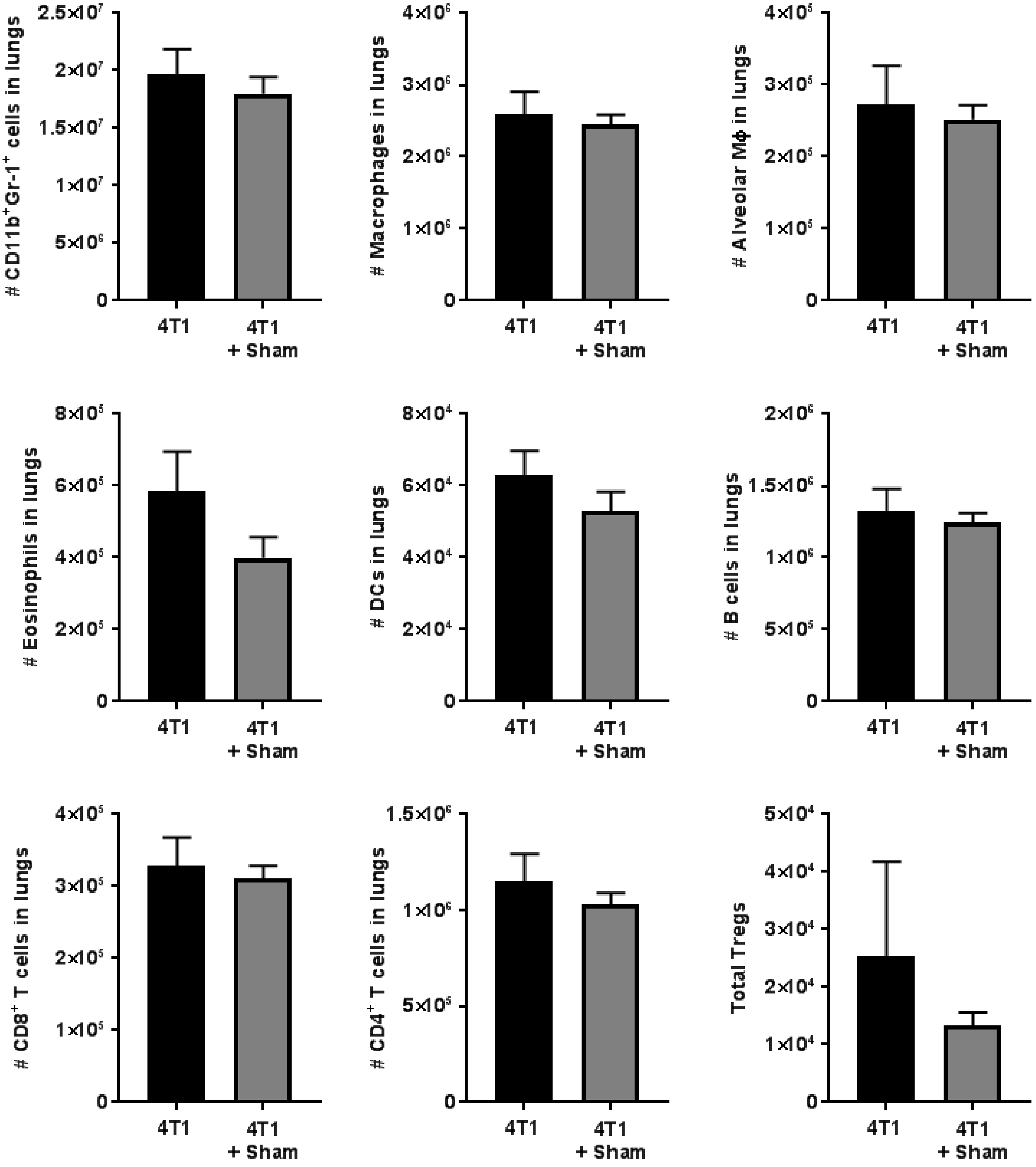

Supplemental Figure 9: **A)** Total number of CD11b$^+$Gr-1$^+$, macrophages, alveolar macrophages, eosinophils, dendritic cells (DCs), B cells, CD8$^+$ T cells, CD4$^+$ T cells, and T regulatory cells the lungs of 4T1 tumor-bearing mice and 4T1 tumor-bearing mice who underwent a sham surgery.
