# Supplementary material for: Targeting myeloid-derived suppressor cells in combination with primary mammary tumor resection reduces metastatic growth in the lungs
Source: Breast Cancer Res. 2019 Sep 5;21:103. doi: 10.1186/s13058-019-1189-x (PMC6727565; doi:10.1186/s13058-019-1189-x)
Supplement: Supplementary file 10 — Figure S9. Total numbers of CD11b+Gr1+ MDSCs, macrophages, alveolar macrophages, eosinophils, dendritic cells (DCs), B cells, CD8+ T cells, CD4+ T cells, and regulatory T cells (Tregs) in the lungs of 4T1 tumor-bearing mice and 4T1 tumor-bearing mice after sham surgery. None of the comparisons were significantly different. (PDF 122 kb) [file 13058_2019_1189_MOESM10_ESM.pdf]

Supplemental Figure 9

A

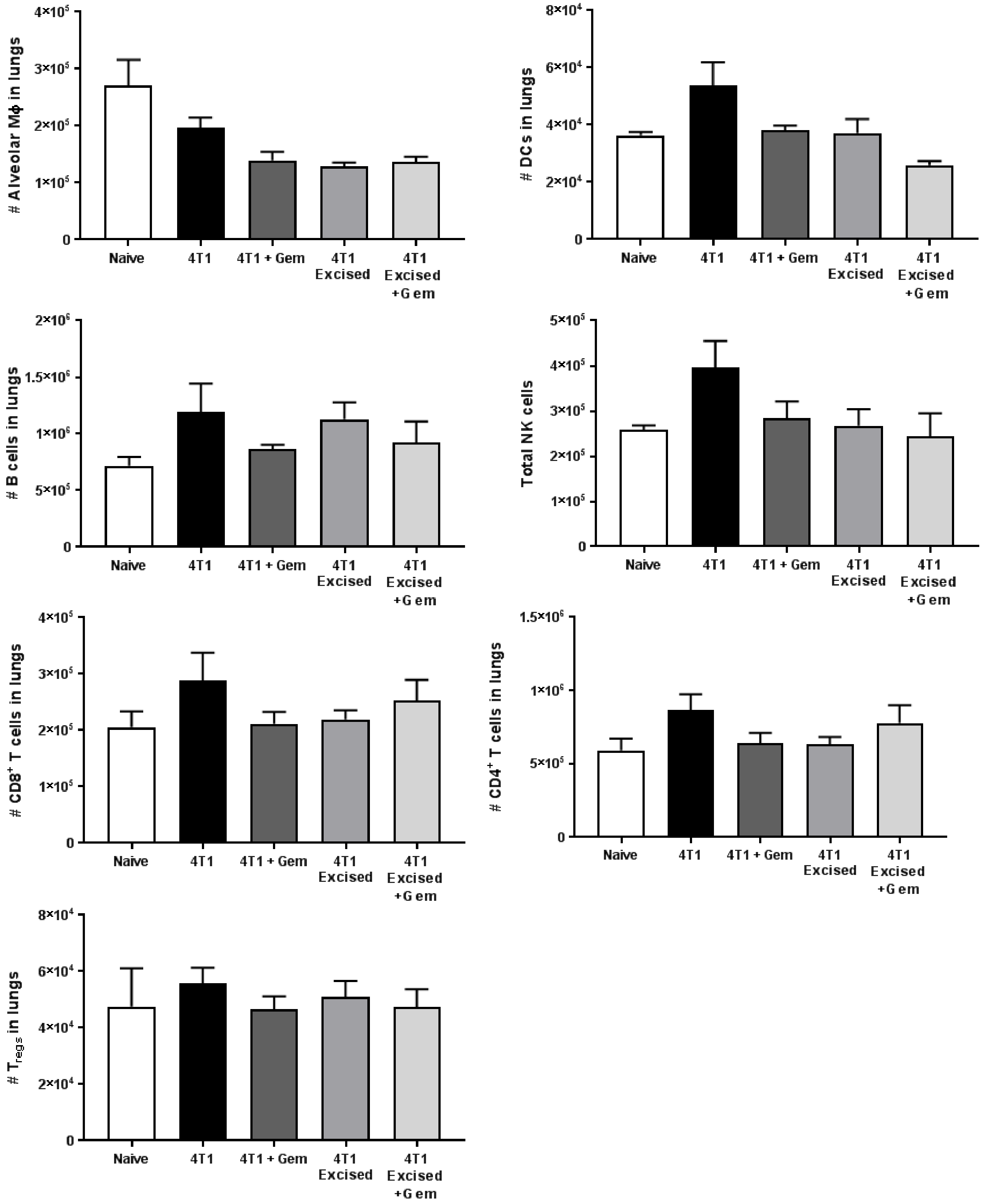

Supplemental Figure 10: **A)** Total number of lung dendritic cells (DCs), alveolar macrophages, B cells, NK cells, $CD8^+$ T cells, $CD4^+$ T cells, and T regulatory cells in mice from experiment outlined in Figure 5A-B.
