# Supplementary material for: Targeting myeloid-derived suppressor cells in combination with primary mammary tumor resection reduces metastatic growth in the lungs
Source: Breast Cancer Res. 2019 Sep 5;21:103. doi: 10.1186/s13058-019-1189-x (PMC6727565; doi:10.1186/s13058-019-1189-x)
Supplement: Supplementary file 11 — Figure S10. A) Total numbers of alveolar macrophages, dendritic cells (DCs), B cells, NK cells, CD8+ T cells, CD4+ T cells, and regulatory T cells (Tregs) in the lungs of mice from the experiment outlined in Fig. 5. None of the comparisons were significantly different. (PDF 99 kb) [file 13058_2019_1189_MOESM11_ESM.pdf]

Supplemental Figure 10

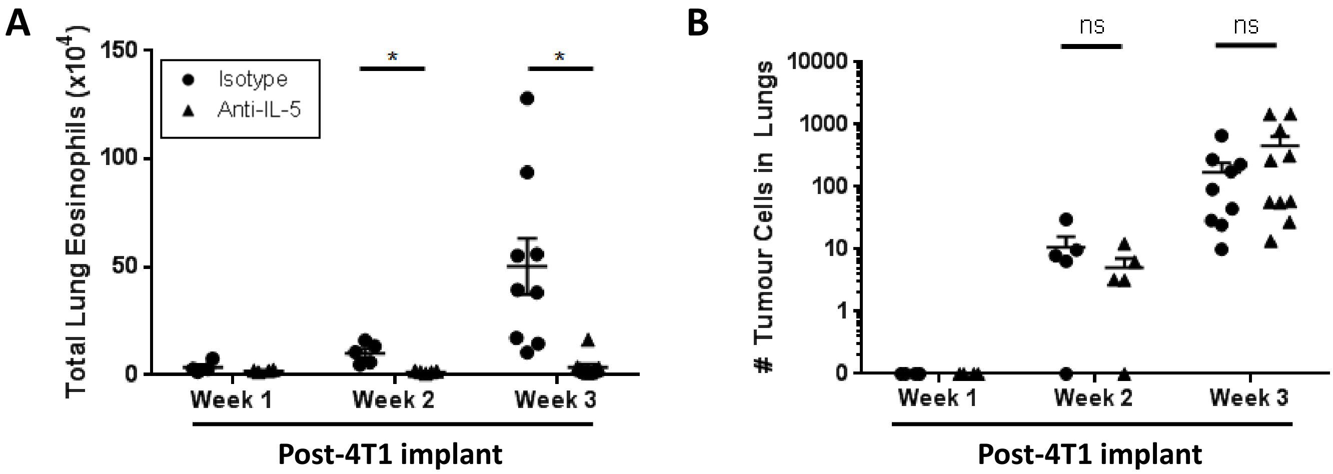

Supplemental Figure 11: **A)** Total number of lung eosinophils in 4T1 tumor-bearing mice treated with anti-IL5 antibody (clone TRFK5) or isotype control. **B)** Number of 4T1 cells in the lungs of mice implanted with orthotopic 4T1 tumors and treated with anti-IL5 antibody or isotype control.
