# Supplementary material for: Targeting myeloid-derived suppressor cells in combination with primary mammary tumor resection reduces metastatic growth in the lungs
Source: Breast Cancer Res. 2019 Sep 5;21:103. doi: 10.1186/s13058-019-1189-x (PMC6727565; doi:10.1186/s13058-019-1189-x)
Supplement: Supplementary file 12 — Figure S11. A) Numbers of eosinophils in the lungs of 4T1 tumor-bearing mice treated with anti-IL5 antibody (clone TRFK5) or isotype control. B) Numbers of 4T1 tumor cells in the lungs of 4T1 tumor-bearing mice treated with anti-IL5 antibody or isotype control. Data are mean ± SEM with n = 4–10 mice per group. (PDF 241 kb) [file 13058_2019_1189_MOESM12_ESM.pdf]

Supplemental Figure 11

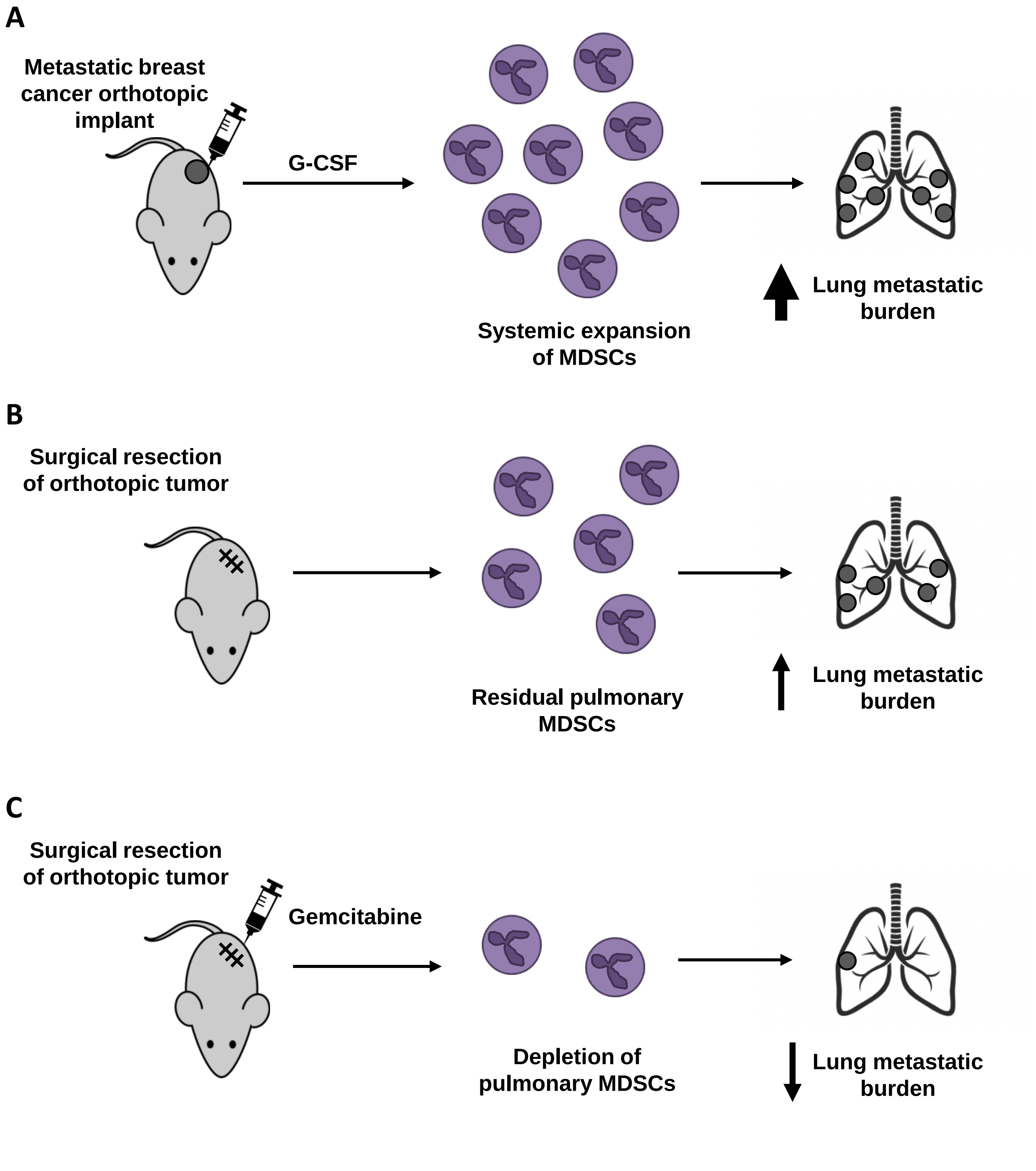

Supplemental Figure 12: Schematic of major findings **A)** Metastatic breast cancer cells secrete G-CSF, driving a systemic expansion of MDSCs and increased lung metastatic burden. **B)** Residual MDSCs continue to support lung metastatic colonization following surgical resection of orthotopic breast cancer tumors. **C)** Combination of surgical resection and low-dose gemcitabine treatment depletes pulmonary MDSCs and decreases lung metastatic burden.
